# Temperature and developmental stage govern intestinal susceptibility to human coronavirus 229E

**DOI:** 10.1073/pnas.2600632123

**Published:** 2026-06-24

**Authors:** Aleksandra Synowiec, Laurensius Kevin Lie, Katarzyna Owczarek, Nina Johannesson, Nina Mickiewicz, Heng-Chang Chen, Artur Szczepański, Madison S. Strine, Renata B. Filler, Maciej Borowiec, Michał Pietrusiński, Izabela Dróżdż, Agnieszka Robaszkiewicz, Michał Bochenek, Matthias Zilbauer, Justyna Rymarowicz, Michał Pędziwiatr, Liza Konnikova, Craig B. Wilen, Dasja Pajkrt, Katja C. Wolthers, Carlemi Calitz, Adithya Sridhar, Krzysztof Pyrc

**Affiliations:** ^a^ Virogenetics Laboratory of Virology, Małopolska Centre of Biotechnology, Jagiellonian University, Kraków 30-387, Poland; ^b^ Doctoral School of Exact and Natural Sciences, Jagiellonian University, Kraków 30-348, Poland; ^c^Department of Laboratory Medicine, Yale School of Medicine, New Haven, CT 06510; ^d^Department of Immunobiology, Yale School of Medicine, New Haven, CT 06510; ^e^ OrganoVIR Labs, Emma Children’s Hospital, Department of Pediatric Infectious Diseases, Amsterdam University Medical Center, Academic Medical Center, Amsterdam Institute for Infection and Immunity, Amsterdam Institute for Reproduction and Development, University of Amsterdam, Amsterdam 1105 AZ, The Netherlands; ^f^ OrganoVIR Labs, Department of Medical Microbiology, Amsterdam University Medical Center, Academic Medical Center, Amsterdam Institute for Infection and Immunity, University of Amsterdam, Amsterdam 1105 AZ, The Netherlands; ^g^ Microbiology Department, Faculty of Biochemistry, Biophysics and Biotechnology, Jagiellonian University, Kraków 30-387, Poland; ^h^Quantitative Virology Research Group, Population Diagnostics Center, Łukasiewicz Research Network–PORT Polish Center for Technology Development, Wrocław 54-066, Poland; ^i^ Department of Clinical and Laboratory Genetics, Medical University of Lodz, Lodz 92-213, Poland; ^j^ Department of Clinical Genetics, Medical University of Lodz, Lodz 92-213, Poland; ^k^ Department of General Biophysics, Faculty of Biology and Environmental Protection, University of Lodz, Lodz 90-236, Poland; ^l^ Department of Pediatrics, School of Clinical Medicine, University of Cambridge, Cambridge CB2 0QQ, United Kingdom; ^m^ Department of General Surgery, Jagiellonian University Medical College, Kraków 30-688, Poland; ^n^ Emma Center for Personalized Medicine, Amsterdam UMC, Amsterdam 1105 AZ, The Netherlands

**Keywords:** coronavirus, HCoV-229E, viral tropism, human intestinal enteroids, host restriction

## Abstract

Human coronaviruses have been primarily associated with upper respiratory tract infections, yet cases of gastrointestinal symptoms in COVID-19 patients have highlighted their potential to cause systemic disease. Here, we detail the infection of intestinal epithelia by an endemic, low-pathogenic human coronavirus, human alphacoronavirus 229E, using patient-derived human intestinal enteroids (HIEs) from donors of various ages. Using fetal, pediatric, and adult HIEs, we investigated how physiologically relevant temperatures: 37 °C and 32 °C, reflecting gastrointestinal and upper-airway conditions, respectively, modulate epithelial responses and viral infection dynamics. We show that there is temperature-dependent transcriptional reprogramming, indicating strong temperature-dependent regulation of virus replication and epithelial responses. Among the seasonal coronaviruses tested, only HCoV-229E productively infects HIEs. At 32 °C, HCoV-229E replicates efficiently in enteroids from all donor ages and releases high titers of infectious progeny. In contrast, at 37 °C, productive replication is largely confined to fetal and a subset of pediatric tissues, revealing a developmental and temperature-sensitive restriction on infection. Confocal and flow cytometry analyses identify enterocytes as the primary target cells for HCoV-229E. Furthermore, we show that camostat, a serine protease inhibitor, significantly reduces HCoV-229E replication in HIEs, confirming a critical role for host serine protease activity. Collectively, these findings establish HIEs as a relevant model for HCoV-229E–host interactions and reveal temperature- and age-dependent determinants governing intestinal permissiveness to this seasonal coronavirus.

## Significance

While human coronaviruses are traditionally viewed as upper respiratory pathogens, the COVID-19 pandemic underscored their potential to cause gastrointestinal disease. This study provides detailed characterization of intestinal infection by the endemic human coronavirus 229E (HCoV-229E) using patient-derived human intestinal enteroids. We reveal that the intestine is not merely a passive bystander but a potential reservoir for infection, governed strictly by physiological barriers. We demonstrate a unique temperature- and age-dependent restriction: While the virus replicates efficiently at upper-airway temperatures across all ages, core-body temperatures restrict replication primarily to fetal and pediatric tissues. These findings uncover a developmental mechanism of host resistance in adults and establish a critical model for testing therapeutics.

Coronaviruses are enveloped, positive-sense, single-stranded RNA viruses with a wide range of vertebrate hosts. Of the seven human coronaviruses (HCoVs), OC43, HKU1, NL63, and 229E are endemic and typically cause mild to moderate respiratory symptoms in the general population (1). However, more severe cases associated with these low-pathogenic HCoVs have been reported in the elderly, children, and immunocompromised (2–5). The remaining three HCoVs are highly pathogenic and responsible for outbreaks or pandemics of severe acute respiratory syndrome over the past 20 y. These include SARS-CoV, MERS-CoV, and SARS-CoV-2 (6–8). Low-pathogenic HCoVs typically infect the upper airway tract, where temperatures are lower (1, 9, 10). Therefore, they adapted and current strains replicate most efficiently at temperatures not exceeding 32 to 34 °C (9, 10). At the same time, newly emerged strains of highly pathogenic HCoVs also replicate effectively at 37 °C, the temperature of the lower respiratory tract (11–13). HCoVs have primarily been associated with respiratory tract illness; however, extrapulmonary clinical manifestations have also been reported (14). For instance, there have been multiple cases of patients with SARS-CoV, MERS-CoV, or SARS-CoV-2 infections presenting hepatic (15–17), cardiovascular (18–20), and neurological (21–23) complications. Another extrapulmonary site of HCoVs infection is the gastrointestinal (GI) tract, as SARS-CoV, MERS-CoV, and SARS-CoV-2 patients presented GI symptoms, accompanied by the presence of the virus in feces (24–27). Although seasonal HCoVs are also viewed as respiratory pathogens, diarrheal outbreaks have been reported, particularly in neonates and young children living in low-resource settings with poor sanitation conditions (28–30). Similarly, GI symptoms and detection of viral RNA in stools have been reported in patients with upper respiratory tract infection caused by seasonal, low-pathogenic HCoVs (31–33). Further, the enteric tropism is well established in multiple nonhuman mammalian coronaviruses, including transmissible gastroenteritis virus (34) or porcine epidemic diarrhea virus (PEDV) in pigs (35), bovine coronavirus in cattle (36), and feline enteric coronavirus (FECV) in cats (37). These natural enterotropic infections highlight that coronaviruses possess an inherent capacity to exploit intestinal epithelial niches, supporting the biological plausibility that seasonal HCoVs may infect the gut under permissive conditions.

The limited understanding of the HCoVs pathogenesis in the GI tract is partially related to the fact that experimental models more closely recapitulating the in vivo physiology of human tissues have become available only recently. Patient-derived human intestinal enteroids (HIEs) (38–40) are more effective at reconstructing morphological and functional properties of in vivo intestinal epithelia than cell lines (38, 41), as they better reflect the physiological characteristics of the infection microenvironment. HIEs are formed using patient-isolated LGR5^+^ intestinal stem cells, localized near Paneth cells at the intestinal crypt domain, which continuously divide and differentiate into major cell types that form the intestinal epithelium (41–43). These include enterocytes, enteroendocrine cells (EECs), goblet, Paneth, and tuft cells, which self-assemble to form the mature epithelial structure (38, 41, 43). Remarkably, HIEs differ in their transcriptional, morphological, and functional properties across age groups (44). For example, morphologically, infant HIEs have significantly shorter epithelial cell height and reduced barrier integrity, and functionally, they mount weaker innate immune responses than adult HIEs (44). HIEs derived from adult, children, or fetal stem cells have been used to characterize infection by enteric viruses such as norovirus (40, 45–47), rotavirus (39, 48), or enterovirus (49, 50). Furthermore, HIEs also supported SARS-CoV (51), MERS-CoV (26), and SARS-CoV-2 (47, 51–53) replication, indicating intestinal epithelia as an extrapulmonary site of infection for these highly pathogenic HCoVs.

Here, using fetal, pediatric, and adult HIEs, we demonstrate that mimicking the physiological temperatures of the upper respiratory (32 °C) and gastrointestinal (37 °C) tracts in HIEs markedly reshape epithelial transcriptional profiles. Among the seasonal human coronaviruses tested, only HCoV-229E productively infects HIEs, revealing the capacity of a respiratory virus to exploit the intestinal epithelium. At 32 °C, HCoV-229E replicates robustly in HIEs across all donor ages and yields abundant infectious progeny. In contrast, at 37 °C, productive replication is largely restricted to fetal and, to a lesser extent, pediatric tissues, indicating a developmental constraint on temperature-tolerant infection. Enterocytes were identified as the main target cell type, consistent with the epithelial distribution of viral entry factors. Finally, inhibition of serine proteases with camostat markedly reduces viral replication. Together, these findings position HIEs as a relevant model for dissecting HCoV-229E–host interactions and reveal temperature and age-dependent determinants that may enable a seasonal coronavirus access extrapulmonary sites.

## Results

### Temperature-Dependent and Age-Dependent Reprogramming of Metabolic and Translational Pathways in HIEs.

To investigate the effects of temperature on the coronaviral replication, we first established a transwell model of differentiated HIEs derived from three age groups: fetal (fHIEs), pediatric (pHIEs), and adult (aHIEs). The differentiation of HIEs was monitored by means of transepithelial electrical resistance (TEER), a quantitative indicator of epithelial barrier integrity and tight junction formation (54). Upon differentiation, TEER values increased progressively over time, exhibiting donor-to-donor variability, and exceeding 200 Ω/cm^2^ (*SI Appendix*, Fig. S1 *A*–*H*), consistent with the formation of a mature, polarized epithelium. To compare the epithelial barrier integrity across developmental stages, TEER was measured in fetal, pediatric, and adult HIE monolayers at the final day of differentiation (Day 13) for all donors (*SI Appendix*, Fig. S1*I*). TEER values from individual donors were pooled within each age group and compared across groups (*SI Appendix*, Fig. S1*J*). Adult HIEs exhibited the highest TEER, followed by pediatric and fetal HIEs, which showed progressively lower values. All pairwise differences were statistically significant (*P* < 0.0001). These results indicate that fetal HIEs have the most permeable epithelial barrier, whereas adult HIEs form the tightest monolayers, with pediatric lines displaying intermediate integrity.

**Fig. 1.**
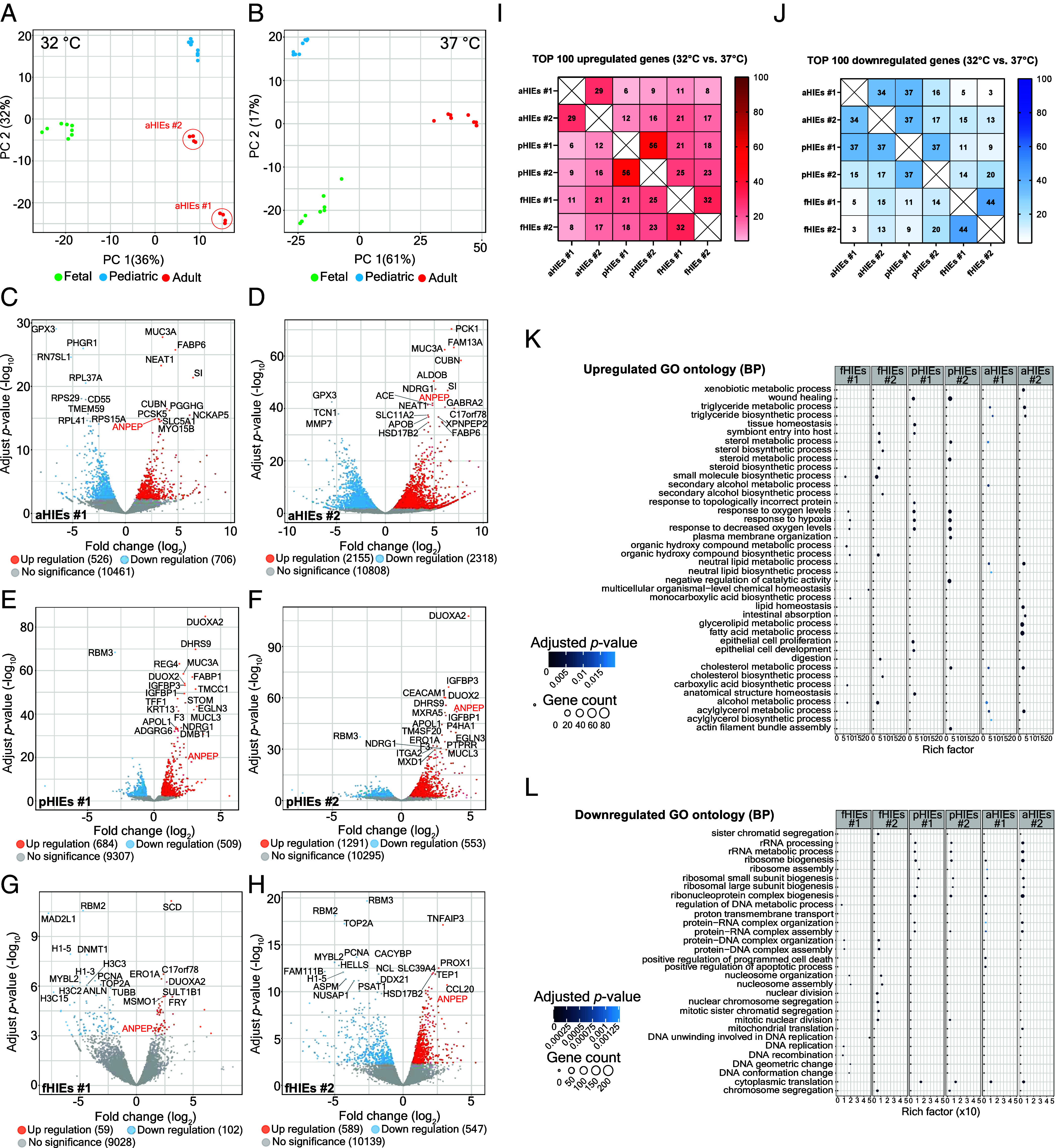
Temperature- and age-dependent transcriptional changes in HIEs analyzed by RNA-seq. (*A* and *B*) PCA of samples from three age groups (two donors per age group) cultured at 32 °C (*A*) or 37 °C (*B*), showing segregation by age (PC1) and temperature (PC2). (*C*–*H*) Volcano plots of DEGs for adult (*C* and *D*), pediatric (*E* and *F*), and fetal (*G* and *H*) datasets. Parentheses placed after the panel legend indicate the number of upregulated and downregulated DEGs, and those are not significantly enriched. (*I* and *J*) Heatmaps depicting the differential expression (log^2^ fold change) of the top 100 upregulated (*I*) and top 100 downregulated (*J*) genes at 37 °C compared to 32 °C. (*K* and *L*) Gene ontology (GO) enrichment analysis showing top upregulated (*K*) and downregulated (*L*) pathways across different donors. Bubble plot showing significantly enriched GO biological process terms for multiple donors. The size of each bubble indicates the number of genes associated with the GO term, while the color distinguishes between experimental groups. Only terms with significant enrichment (adjusted *P* < 0.05) are shown.

To explore how temperature influences gene expression, we performed bulk RNA sequencing (RNA-seq) to compare the transcriptional profiles of HIEs cultured at 32 and 37 °C. Principal component analysis (PCA) demonstrated distinct age-related clustering of HIEs at both temperatures (Fig. 1 *A* and *B*), confirming global transcriptomic age-related differences. Differential gene expression (DEG) analysis for adult (Fig. 1 *C* and *D*), pediatric (Fig. 1 *E* and *F*), and fetal (Fig. 1 *G* and *H*) donors further quantified these temperature-driven changes across age groups. Volcano plots revealed a robust set of genes significantly up- and down-regulated at 32 °C compared to 37 °C, with both the number and magnitude of DEGs varying among age groups. While certain genes showed consistent regulation across all conditions, others displayed marked age-specific effects, underscoring a dynamic interplay between host age and environmental temperature in shaping transcriptional changes. To identify common and distinct transcriptional patterns across ages, we next examined the top 100 upregulated (Fig. 1*I*) and the top 100 downregulated (Fig. 1*J*) genes for each age group and compared their overlap. This comparison revealed that a subset of temperature-dependent genes was shared across all ages, such as upregulation of aminopeptidase N (*ANPEP*) (an entry receptor for HCoV-229E), and aldolase (*ALDOB*), or downregulation of RNA-binding protein 3 (*RBM3*) and beta tubulin (*TUBB*). Nevertheless, many genes exhibited age-specific expression changes. To gain functional insight into the observed transcriptional changes, we performed gene ontology biological process (GOBP) enrichment analysis on the identified DEGs and analyzed the most upregulated (Fig. 1*K*) and downregulated (Fig. 1*L*) pathways. At 37 °C, analysis revealed a strong activation of metabolic and hypoxia-associated pathways. The most significantly enriched terms for upregulated genes included: organic acid metabolic process (*GO:0006082*), carbohydrate catabolic process (*GO:0016052*), carboxylic acid metabolic process (*GO:0019752*), and small molecule metabolic and biosynthetic processes (*GO:0044281, GO:0044283*), indicating a global reprogramming of cellular metabolism toward increased catabolic and biosynthetic activity. Notably, several terms related to lipid metabolism include lipid metabolic process (*GO:0006629*), acylglycerol metabolic process (*GO:0006639*), neutral lipid metabolic process (*GO:0006638*), and sterol biosynthetic process (*GO:0016126*). Enrichment of these pathways suggests enhanced lipid remodeling and sterol synthesis at higher temperatures. In addition, upregulated genes were associated with pathways linked to oxygen sensing and hypoxic adaptation, such as response to hypoxia (*GO:0001666*), response to decreased oxygen levels (*GO:0036293*), and response to oxygen levels (*GO:0070482*), together with cholesterol and sterol homeostasis (*GO:0042632, GO:0055092*). These enrichments imply that a temperature of 37 °C triggers metabolic and homeostatic adjustments resembling those activated under reduced oxygen availability, consistent with increased energetic and membrane demands at this temperature. Collectively, these results highlight that higher temperature promotes a coordinated transcriptional program centered on lipid metabolism, energy production, and hypoxia-responsive processes. Notably, the adult group did not display upregulation of many stress-response and cell–cell adhesion-related genes at 37 °C, indicating an age-dependent modulation of cellular stress adaptation to temperature. A heatmap of pathway-associated genes further illustrated these age- and temperature-dependent differences for cell adhesion (*SI Appendix*, Fig. S2*A*) and stress response pathways (*SI Appendix*, Fig. S2*B*).

In contrast, downregulated genes at 37 °C were predominantly associated with cytoplasmic translation (*GO:0002181*) and ribosomal small unit assembly (*GO:0000028*), indicating a global reduction in protein synthesis compared to 32 °C. The downregulation of these pathways was consistently observed across all age groups, with the most pronounced effect in adult donors, suggesting age-dependent differences in the ability to maintain translational activity at elevated temperatures. This age-dependent reduction in translational activity contrasts with the upregulation of lipid metabolism and stress-response genes at 37 °C, indicating a reciprocal regulation between protein synthesis and adaptive metabolic processes. These findings suggest that higher temperature induces a shift in cellular resources from growth and translation toward protective metabolic programs in an age-dependent manner. A heatmap of cytoplasmic translation pathway-associated genes further illustrated this age-dependent suppression (*SI Appendix*, Fig. S2*D*). In addition to the suppression of cytoplasmic translation, our RNA-seq analysis of HIEs revealed significant age-dependent transcriptional shifts in genes encoding the Nop56-associated pre-rRNA complex and the spliceosome C-complex. While previous structural proteomics indicated that these complexes undergo major conformational changes during coronavirus infection to facilitate host-cell hijacking (55), our data demonstrate a corresponding thermal modulation at the mRNA level (*SI Appendix*, Fig. S2*C*). We have also checked the baseline expression levels for the cytoplasmic translation genes (*SI Appendix*, Fig. S3) as well as for the Nop56-associated pre-rRNA complex and the spliceosome C-complex (*SI Appendix*, Fig. S4).

### Temperature-Dependent HCoV-229E Infection of HIEs Derived from Donors of Different Age Groups.

**Fig. 2.**
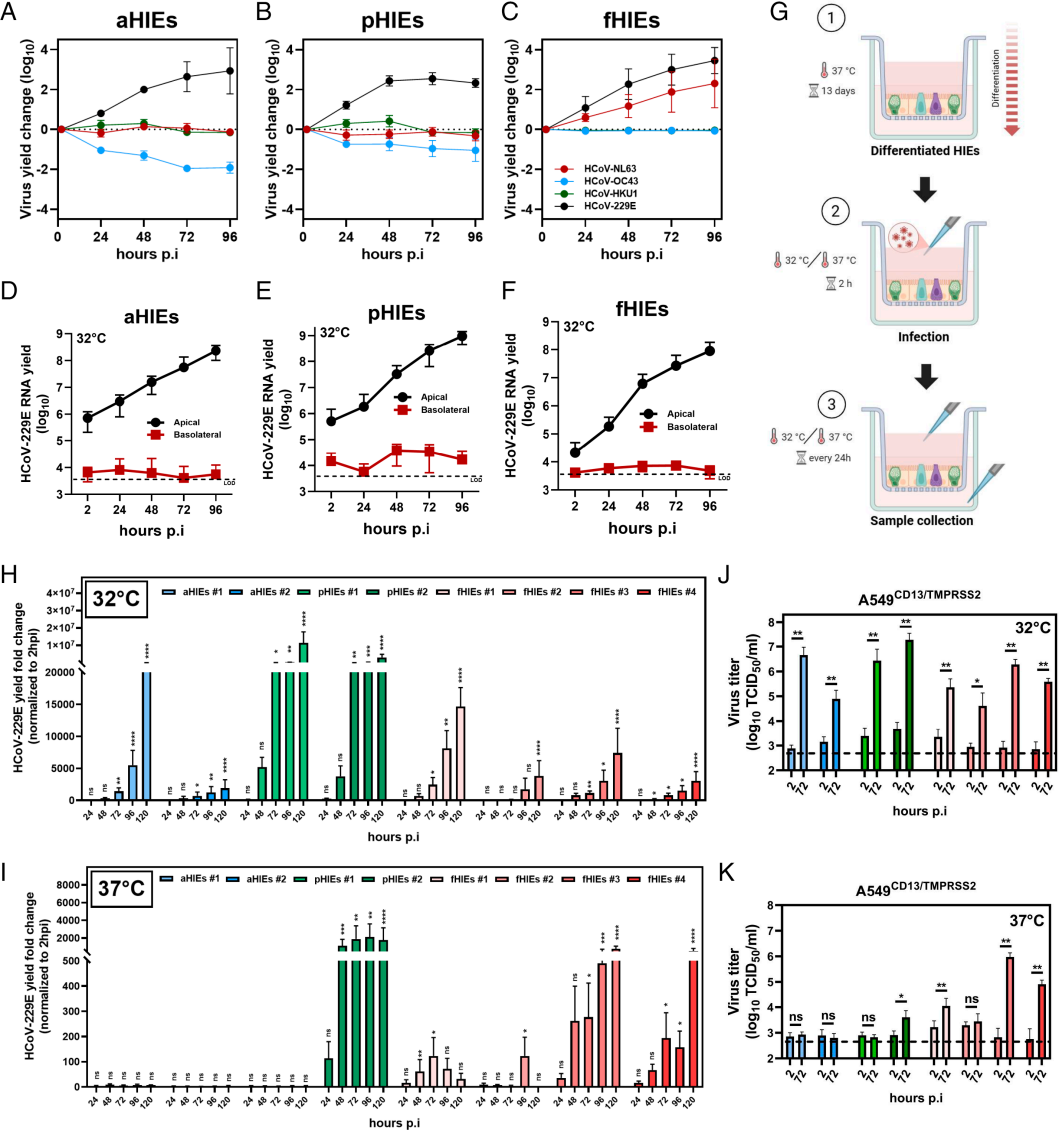
Replication of seasonal, low-pathogenic HCoVs in differentiated HIEs. (*A*–*C*) Infection of (*A*) adult (aHIEs #1), (*B*) pediatric (pHIEs #2), and (*C*) fetal HIEs (fHIEs #1) at 32 °C with HCoV-NL63, HCoV-OC43, HCoV-HKU1, and HCoV-229E up to 96 h p.i. Samples were normalized to 2 h p.i. and presented as log_10_ change of virus yield. The dashed line represents zero-fold change. (*D*–*F*) Apical and basolateral supernatants from HCoV-229E–infected HIEs were collected at the indicated time points and analyzed by RT-qPCR to quantify viral RNA yield (RNA copies/transwell). Viral RNA was detected exclusively in the apical compartment, with no increase observed at the basolateral surface in any donor, indicating that viral release occurs apically. (*G*) The experimental setup for viral infection of HIEs in the transwell system is schematically illustrated. (*H*–*K*) Replication of HCoV-229E in eight different donors (*H* and *J*) at 32 °C or (*I* and *K*) at 37 °C. Data are presented as a fold change of virus yield (compared to 2 h p.i.). (*H* and *I*) and virus titer (log_10_ TCID_50_/mL) HIEs supernatants at 32 °C using A549^CD13/TMPRSS2^ (*J* and *K*). Supernatants at 2 h p.i. and 72 h p.i. were collected from the apical site of infected HIEs from eight different donors. The dashed line represents the limit of detection. (*B*–*K*) Data are acquired from at least two independent experiments performed in triplicate. Statistical analyses were performed using the (*H* and *I*) Friedman Test (Dunn’s corrected) or (*J* and *K*) two-tailed Mann–Whitney U-Test on non-log-transformed data. Data are presented as mean ± SEM or median ± IQR. “ns” = no statistical significance, *P < 0.05, **P < 0.01, ***P < 0.001, ****P < 0.0001.

To determine whether seasonal, low-pathogenic HCoVs can infect the intestinal epithelium, we examined their ability to infect differentiated HIEs generated from fetal, pediatric, and adult donors. While fetal intestinal infection is atypical, evaluating viral replication in this developmentally unique environment clarifies whether epithelial maturity or specific progenitor populations dictate viral permissiveness. Differentiated monolayers were apically inoculated with a 10× diluted infectious stock of clinical strains of HCoV-229E, HCoV-NL63, HCoV-OC43, or HCoV-HKU1 generated in human airway epithelial (HAE) cultures. Both inoculation and incubation were performed at 32 °C to simulate the ambient temperature of the upper respiratory tract. Supernatants were collected from the apical side at 2 h postinfection (p.i.), then every 24 h, to assess changes in viral RNA content in the supernatant throughout infection. qPCR analyses of the apical supernatants revealed that HCoV-229E exhibited an increase in viral RNA levels over the incubation period across all the HIE donors (Fig. 2 *A*–*C*). Notably, HCoV-NL63 also demonstrated an increase in viral RNA, but it was restricted only to fHIEs. In contrast, no detectable increase in viral RNA was observed for HCoV-OC43 or HCoV-HKU1 (Fig. 2 *A*–*C*). As efficient replication was observed exclusively for HCoV-229E, following experiments were therefore focused on this coronavirus. Subsequently, supernatants were collected from both the apical and basolateral compartments following infection to determine the site of infectious virus release. HCoV-229E RNA levels increased only in the apical compartment, with no detectable rise in the basolateral site (Fig. 2 *D*–*F*), indicating that virus progenies are released exclusively to the apical surface of HIEs. Next, we asked whether the observed infection pattern was influenced by age group, donor variability, and/or temperature, and for this purpose, we expanded our HIEs panel to include additional donors. In total, HIEs were derived from two adult (aHIEs #1, aHIEs #2), two pediatric (pHIEs #1, pHIEs #2), and four fetal (fHIEs #1, fHIEs #2, fHIEs #3, and fHIEs #4) donors.

All HIEs were infected with HCoV-229E and incubated at either 32 °C, mimicking the physiological temperature of the upper respiratory tract, or 37 °C, corresponding to that of the physiological temperature of the GI tract. The experimental setup for viral infection of HIEs in the transwell system is schematically illustrated (Fig. 2*G*). RT-qPCR analysis confirmed an increase in the number of HCoV-229E RNA copies in apical supernatants from all age groups at 32 °C (Fig. 2*H*). A similar trend was observed at 37 °C, although this was limited to fHIEs and one pediatric donor (pHIEs #2) (Fig. 2*I*). Additionally, supernatants from infected HIEs were collected and titrated using permissive A549^CD13/TMPRSS2^ cell line (56) to assess whether produced in HIEs viral progenies are infectious. Accordingly, cytopathic effect (CPE) was detected in the cell line treated with supernatants from all HIEs donors at 32 °C (Fig. 2*J*), but only from three fetal and one pediatric HIEs donors at 37 °C (Fig. 2*K*).

**Fig. 3.**
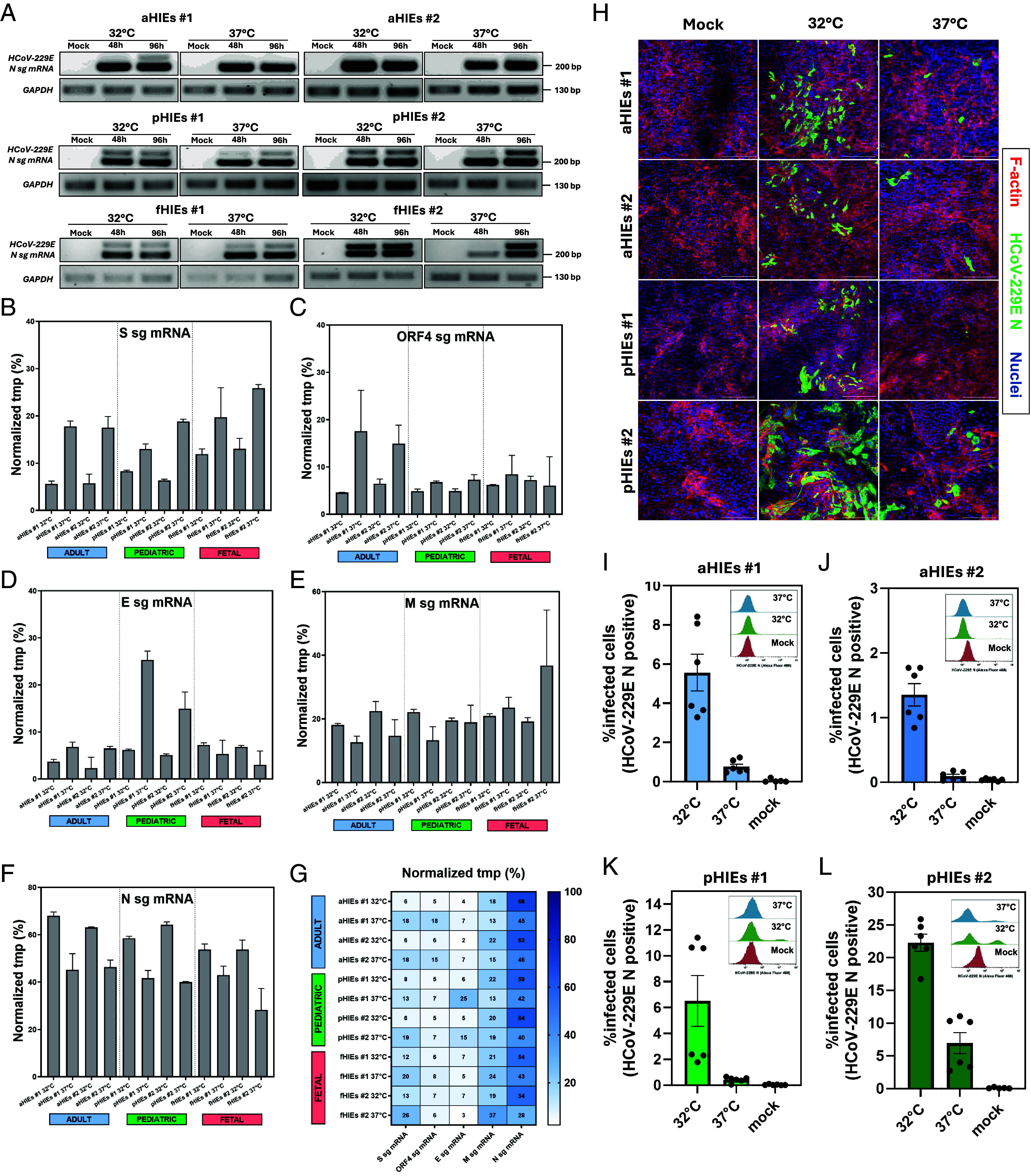
HCoV-229E entry and replication in HIEs. (*A*) Detection of HCoV-229E nucleocapsid (N) sg mRNA in differentiated HIEs derived from adult (aHIEs #1 to 2), pediatric (pHIEs #1 to 2), and fetal (fHIEs #1 to 4) donors. HIEs were infected apically with HCoV-229E and incubated at either 32 or 37 °C for 48 or 96 h. cDNA from infected monolayers was subjected to seminested PCR amplification of sg N mRNA, and products were visualized on 2.5% agarose gels. GAPDH mRNA served as a loading control. The presence of N sg mRNA confirms active viral replication. (*B*–*G*) HCoV-229E subgenomic mRNA expression profiles determined by RNA-seq. Relative abundance of viral subgenomic mRNAs (sg mRNA) of (*B*) spike (S), (*C*) ORF4, (*D*) envelope (E), (*E*) membrane (M), and (*F*) nucleocapsid (N), shown as transcript mapping percentage (tmp, %) in HIEs derived from different donors and cultured at either 32 or 37 °C. Data represent mean ± SEM from two independent biological replicates. (*G*) Heatmap summarizing viral transcript expression patterns from panels *A* to *E*, highlighting overall similarities and differences in transcript abundance across donors and temperatures. (*H*) Representative immunofluorescence images of HCoV-229E-infected HIEs at 48 h p.i. at 32 or 37 °C. Viral N protein is shown in green, F-actin in red, and nuclei in blue. Viral spread and the number of infected cells were markedly reduced at 37 °C compared to 32 °C across all donors. (*I*–*L*) Quantification of HCoV-229E-infected cells by flow cytometry in adult (aHIEs #1 to 2) and pediatric (pHIEs #1 to 2) donors at 48 h p.i. Mock represents the not-infected control. Bars represent the mean ± SD from at least two independent experiments, with representative histograms shown on the right. Data and images were acquired from at least two independent experiments with *n* = 6 per enteroid line. No asterisk = no statistical significance, **P < 0.01. (Scale bar, 50 μm.)

To further confirm active replication of HCoV-229E in HIEs, we performed a seminested PCR targeting the *N* subgenomic mRNA (sg mRNA) of HCoV-229E using the primer set described previously (57). During coronavirus replication, sg mRNAs are produced as intermediates of viral transcription and serve as a hallmark of active viral replication in the host. Fully differentiated aHIEs, pHIEs, and fHIEs were apically inoculated with 1.43 × 10^4^ TCID_50_/mL of HCoV-229E and incubated for 2 h at either 32 or 37 °C, followed by incubation at the respective temperatures after the removal of unbound virus. PCR amplification of an N-gene fragment from virus-infected cells yielded products of the expected sizes (203 and 280 bp), a prominent band of approximately 200 bp was consistently detected in all infected monolayers, irrespective of incubation temperature, up to 96 h p.i. (Fig. 3*A*), confirming active HCoV-229E replication within all HIEs. Importantly, we did not observe the increase in viral RNA copies in the supernatants or production of infectious viral particles in adult and one pediatric donor at 37 °C (Fig. 1 *I* and *K*). Next, we performed RNA-seq data analysis of infected and noninfected samples that identified robust viral transcriptional activity, with clear detection of multiple viral subgenomic (sg) mRNAs in infected cells (Fig. 3 *B*–*G*). We detected viral sg mRNAs coding for S (Fig. 3*B*), ORF4 (Fig. 3*C*), E (Fig. 3*D*), M (Fig. 3*E*), and N (Fig. 3*F*). Comparative analysis across the two incubation temperatures showed significant differences in the relative abundance of individual sg mRNAs, indicating trends in temperature-dependent regulation of viral gene expression. Moreover, the pattern of sg mRNA accumulation varied between donors, with donor age emerging as an additional factor influencing sg mRNA composition, e.g., ORF4 sg mRNA for adult donors at 37 °C (Fig. 3*C*) or E sg mRNA for pediatric donors at 37 °C (Fig. 3*D*). Altogether, these data suggest that both environmental temperature and host age modulate viral transcriptional dynamics during infection.

To investigate more into the HCoV-229E infection characteristics, HIEs were infected with HCoV-229E and immunostained at 48 h p.i. to assess viral spread (Fig. 3*H*) and quantify the proportion of infected cells (Fig. 3 *I*–*L*). The analysis focused on pediatric and adult HIEs, as fetal HIEs were excluded due to limited material availability. Viral spread was consistently more extensive at 32 °C than at 37 °C across all tested HIEs. Flow cytometry analysis further confirmed these findings, showing that the proportion of infected (N-positive) cells was markedly reduced at 37 °C compared to 32 °C. Across all donors, infection rates ranged from approximately 1 to 6% at 37 °C to 2 to 22% at 32 °C, demonstrating a clear temperature-dependent effect on viral replication. In agreement with RT-qPCR and viral titration data, pediatric donor #2 supported efficient viral replication at both temperatures, indicating a higher overall susceptibility to infection (Fig. 3*L*). These results collectively suggest that lower temperature conditions favor more robust viral propagation in HIEs, with donor-specific factors (such as age) further influencing replication efficiency.

### Transcriptomic and Functional Dissection of Host Factors Controlling HCoV-229E Replication, Tropism, and Entry.

**Fig. 4.**
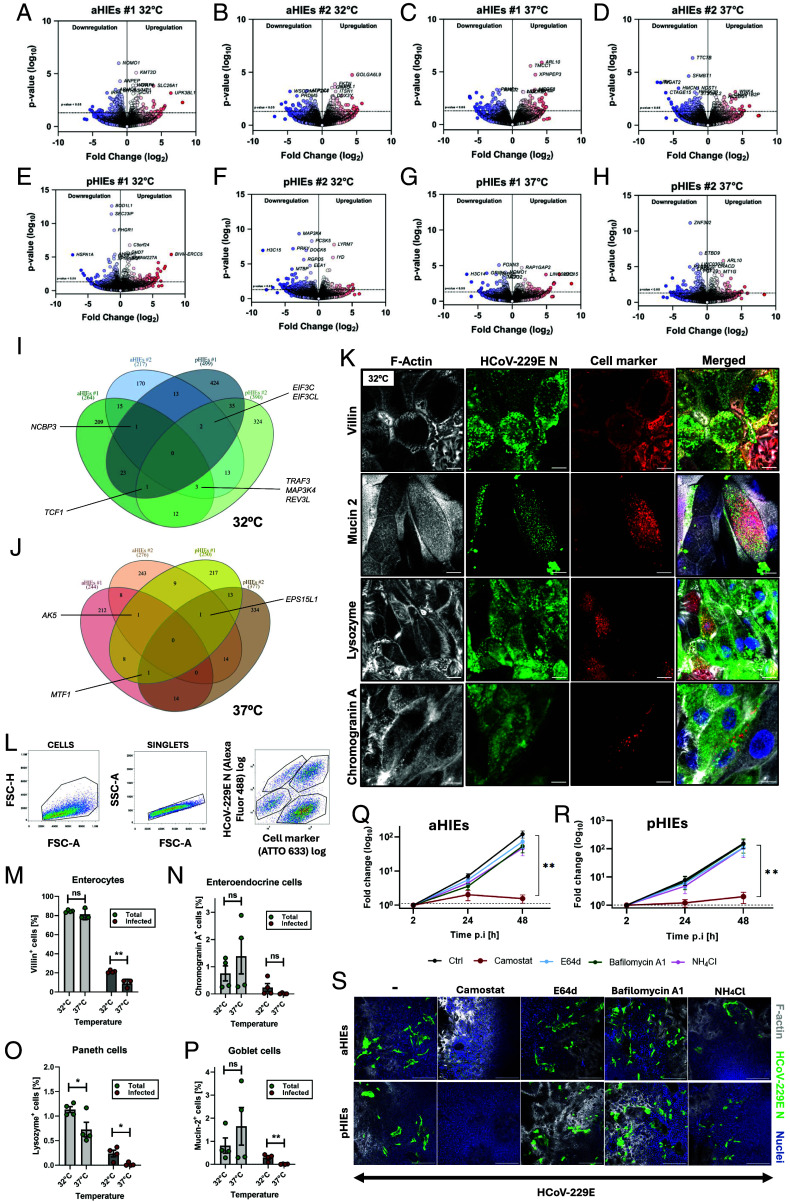
Temperature modulates HCoV-229E cell tropism. (*A*–*H*) Volcano plots of DEGs for not-infected vs HCoV-229E infected adult (*A*–*D*), and pediatric (*E*–*H*) HIEs. (*I* and *J*) Venn diagrams presenting the overlap between DEGs (*P*-value < 0.05) at 32 °C (*I*) or 37 °C (*J*). (*K*) pHIEs monolayers were infected with HCoV-229E and incubated at 32 °C. Subsequently, monolayers were fixed after 48 h p.i. for immunofluorescence staining. Samples were immunostained with antibodies for cell-type-specific markers: villin (enterocytes), mucin-2 (goblet cells), lysozyme (Paneth cells), chromogranin A (enteroendocrine cells), shown in red, alongside HCoV-229E nucleocapsid protein (N) in green. F-actin is shown in gray and nuclei in blue. (Scale bars, 10 µm.) Representative images from two independent experiments are shown. (*L*) gating strategy used for flow cytometry analysis. (*M*–*P*) Quantification of marker-positive and infected cells by flow cytometry: villin (*M*), chromogranin a (*N*), lysozyme (*O*), and mucin-2 (*P*). (*Q* and *R*) Enteroid monolayers were infected with HCoV-229E clinical isolate and maintained in the presence of inhibitors [camostat, E64d, bafilomycin A1, or ammonium chloride (NH_4_Cl)] at 32 °C for 48 h p.i. Graphs represent the increase in viral RNA copies obtained from the supernatant of infected (*Q*) aHIEs or (*R*) pHIEs relative to 2 h p.i. (*S*) Treated enteroid monolayers were then fixed after 48 h p.i and stained for HCoV-229E N. Viral N protein is shown in green, F-actin in gray, and nuclei in blue. (Scale bars, 200 µm.) Data are pooled from two independent experiments and presented as mean ± SEM. Statistical analyses were performed using a two-tailed unpaired Student’s *t* test. ns, not significant, *P < 0.05, **P < 0.01.

To further define host determinants of HCoV-229E susceptibility, we performed bulk RNA-seq on infected (1.43 × 10^5^ TCID_50_/mL) and mock-treated cells at 48 h p.i. We compared the transcriptomic responses of pediatric and adult HIEs following HCoV-229E infection at 32 and 37 °C (Fig. 4 *A*–*H*). Surprisingly, the Venn diagrams revealed poor overlap in significant DEGs across all donors at either temperature (Fig. 4 *I* and *J*), indicating that HCoV-229E elicits highly heterogeneous, condition-dependent host responses. At 32 °C, regulated genes were enriched for RNA-processing and translation-associated factors, including *NCBP3*, *EIF3C*, and *EIF3CL*, consistent with a shift toward temperature-sensitive translational control that accompanies cold-stress-driven changes in RNA handling (58). In contrast, the transcriptional profile at 37 °C prominently involved genes linked to membrane trafficking, endocytosis, and metabolic homeostasis, such as *EPS15L1*, *AK5*, and *MTF1*, reflecting recruitment of a distinct set of temperature-dependent pathways. Among the most significantly modulated genes were *NCBP3*, a component of the alternative cap-binding complex previously implicated in antiviral defense (59), and *TRAF3*, a key adaptor of innate immune signaling (60), both of which were strongly downregulated during HCoV-229E infection but only at 32 °C.

Further transcriptomic analysis of innate immunity responses of all infected HIEs revealed that the universal susceptibility to HCoV-229E infection at 32 °C correlated with a widespread “transcriptional silence” in the innate immune compartment, where critical viral sensors, including *RIG-I* and *IFIH1* (MDA5), as well as downstream effectors like *IFIT1* and *MX1*, showed negligible induction (*SI Appendix*, Fig. S5). Notably, we also did not observe big differences in basal levels of mRNA transcripts associated with antiviral responses (*SI Appendix*, Fig. S6). This is not unexpected, as coronaviruses are well known to attenuate innate immune responses in infected cells. Interestingly, ribonuclease L (*RNASEL*) levels were higher in adult cells than in pediatric and fetal cells, whereas TLR3 expression showed the opposite trend. Therefore, it is important to note the substantial interdonor variability observed across samples.

Given that HCoV-229E replication at 37 °C was notable only in a single pediatric donor (pHIEs #2), we performed comparative transcriptomic profiling to define the molecular determinants of this differential susceptibility. First, we performed comparative transcriptomic profiling of uninfected pHIEs at 32 and 37 °C (*SI Appendix*, Fig. S7). At 37 °C, the susceptible pediatric donor (pHIEs #2) displayed a distinct proviral metabolic signature, characterized by the significant enrichment of purine and ribonucleotide biosynthetic processes. In contrast, the resistant pediatric donor (pHIEs #1) was defined by the upregulation of pathways governing actin filament organization and plasma membrane assembly (Dataset S4). Moreover, the permissive state of pHIEs #2 was further associated with a marked “immunological lag.” Unlike the resistant pHIEs #1, which at 37 °C mounted a robust induction of critical antiviral effectors (*IFIT1*, *IFNA1*, *IFNB1*, *IFNL1, RSAD2*, *ISG15*), pHIEs #2 exhibited a blunted immune profile (*SI Appendix*, Figs. S5 and S6).

To determine the intestinal epithelium cell tropism of HCoV-229E, we focused on HIEs derived from pediatric donor #2 (pHIEs #2), as the enteroids supported efficient viral replication at both 32 and 37 °C. Monolayers were infected with the virus (1.43 × 10^5^ TCID_50_/mL) and incubated for 48 h at either temperature. Infected monolayers were fixed and immunostained for viral N protein and cell-type-specific markers: villin (enterocytes), lysozyme (Paneth cells), mucin-2 (goblet cells), and chromogranin A (EECs). Confocal imaging confirmed the presence of all major epithelial lineages within the cultures (Fig. 4*K*). Colocalization analysis revealed that HCoV-229E predominantly infected villin-positive enterocytes at 32 °C, with occasional infection of goblet cells, Paneth cells, or EECs (Fig. 4*K*). At 37 °C, the colocalization was observed only in enterocytes. Flow cytometry quantification supported these observations (Fig. 4 *I*–*P*). The relative proportions of enterocytes, EECs, and goblet cells were not significantly different between 32 and 37 °C, whereas Paneth cells were significantly less abundant at 37 °C (0.72% ± 0.15%) than at 32 °C (1.14% ± 0.06%). Consistent with confocal data, the majority of infected cells was villin-positive at both temperatures (Fig. 4*M*), indicating that HCoV-229E primarily targets enterocytes. However, at 32 °C, infection of EECs, goblet cells, and Paneth cells was also visible (Fig. 4 *N*–*P*). In contrast, at 37 °C, infection was restricted almost exclusively to enterocytes (Fig. 4 *M*–*P*), demonstrating a temperature-dependent difference in HCoV-229E cell tropism or limited susceptibility of other cell types at lower infection levels.

To delineate the cellular entry mechanism of HCoV-229E in the intestinal epithelium, we infected enteroid monolayers in the presence of known entry inhibitors: camostat mesylate, E64d, bafilomycin A1 (BafA1), and ammonium chloride (NH_4_Cl) (52). The serine protease inhibitor camostat mesylate inhibits serine proteases, e.g., TMPRSS2, which are used by wild-type human coronaviruses for spike priming and enable them for direct entry by fusion with the plasma membrane (56, 61). The cathepsin B/L inhibitor E64d interferes with coronavirus spike processing in the endosome after viral uptake, which takes place in immortalized cell lines during endocytosis, where TMPRSS2 and other similar proteases are not available (62). BafA1 and NH_4_Cl inhibit endosomal acidification and block the endosome-mediated entry of viruses (63). RT-qPCR analyses of culture supernatants showed no suppression of HCoV-229E replication in HIEs treated with E64d, BafA1, or NH_4_Cl. However, HIEs treated with camostat mesylate showed a reduction in viral replication as early as 24 h p.i. (Fig. 4 *Q* and *R*). Furthermore, confocal microscopy analyses showed a reduction in the number of cells positive for HCoV-229E nucleocapsid in camostat-treated enteroid monolayers 48 h p.i, whereas HCoV-229E positive cells were observed abundantly in enteroid monolayers treated with E64d, BafA1, or NH_4_Cl (Fig. 4*S*). Taken together, our data indicate that the HCoV-229E clinical isolate primarily utilizes serine proteases (e.g., TMPRSS2) for cellular entry in the enteroid model at 32 °C.

## Discussion

In this study, we demonstrate that developmental age and temperature jointly determine the susceptibility of the human intestinal epithelium to seasonal human coronaviruses (HCoVs), revealing constraints on enteric viral infection. Although seasonal HCoVs are viewed as respiratory pathogens, diarrheal outbreaks have been reported, particularly in neonates and young children living in low-resource settings with poor sanitation conditions (28–30). Such reports contrast with the overall weak epidemiological association between seasonal HCoVs and GI disease (31), and the fact that a majority of past suspected HCoV-associated gastroenteritis involves a coinfection by bona fide enteric viruses (64, 65). Nonetheless, both low- and high-pathogenic HCoVs have been linked to GI symptoms, and robust evidence exists for SARS-CoV, MERS-CoV, and SARS-CoV-2 (26, 51, 52, 66–68). Further, the enteric tropism is well established in multiple nonhuman mammalian coronaviruses, including transmissible gastroenteritis virus (34) or PEDV in pigs (35), bovine coronavirus in cattle (36), and FECV in cats (37). These natural enterotropic infections highlight that coronaviruses possess an inherent capacity to exploit intestinal epithelial niches, supporting the biological plausibility that seasonal HCoVs may infect the gut under permissive conditions.

Our results show that a clinical isolate of HCoV-229E is uniquely able to productively infect the intestinal epithelium, provided that both the temperature and host developmental stage are favorable. A clinical isolate of HCoV-NL63 showed replication restricted to fetal HIEs, whereas clinical isolates of HCoV-HKU1 and HCoV-OC43 failed to replicate in fetal-, pediatric-, or adult-derived enteroids. HCoV-229E was first isolated in 1966 and is believed to originate from bats with camelids as intermediate hosts (69, 70). HCoV-229E is classified in the genus *Alphacoronavirus.* HCoV-229E utilizes aminopeptidase N (ANPEP; CD13) to enter permissive cells (56, 71). However, like other human respiratory coronaviruses, HCoV-229E also requires priming by serine proteases, possibly transmembrane serine protease 2 (TMPRSS2) (72). While reports of extrapulmonary manifestations of HCoV-229E infection exist, little is known about the molecular mechanisms underlying this process (32, 73).

By using fetal, pediatric, and adult HIEs, we modeled how physiologically relevant temperature niches: 32 °C (upper-airway-like) vs. 37 °C (gastrointestinal) rewire epithelial transcriptional states and, in turn, influence HCoV-229E permissiveness. At 32 °C, HCoV-229E replicates efficiently across all donors, yielding high titers of infectious progeny. In contrast, at 37 °C, replication becomes strongly age-dependent, fetal HIEs remain highly permissive, a subset of pediatric enteroids supports replication, while adult HIEs exhibit marked restriction. These findings suggest that developmental maturation imposes a barrier to viral fitness at physiological gut temperature (37 °C). Also, although aHIEs typically form more robust epithelial barriers than pediatric or fetal cultures, this is unlikely to be the primary driver of HCoV-229E resistance. Since the virus infects enterocytes from the apical surface, susceptibility in this model is more probably determined by developmental maturity and the specific density of the ANPEP receptor. Nevertheless, the generalizability of these findings may be constrained by interdonor variability. Despite the focused sample size within each age bracket, the observed trends remain compelling; however, validating these signatures across larger, high-diversity donor pools is essential.

Transcriptional profiling provides mechanistic insight into the temperature effects. Relative to 32 °C, exposure to 37 °C induced broad downregulation of genes involved in cytoplasmic translation and ribosomal small-subunit assembly, indicating a global reduction in protein synthesis. Mechanistically, this finding may intersect with the replication strategy described by Sarkar et al., who used limited-proteolysis mass-spectrometry (LiP-MS) to show that HCoV-229E hijacks host RNA-processing assemblies, in particular the Nop56-associated pre-rRNA complex and the spliceosome C-complex, and that perturbation of these assemblies inhibits viral replication (55). Thus, temperature-driven translational repression may restrict HCoV-229E access to the ribosome-biogenesis and RNA-processing machinery it exploits, rendering enteroids less permissive at 37 °C by limiting small-subunit assembly and maintaining RNA-processing complexes in less hijackable states.

There are many mechanisms that regulate the temperature dependence of a specific virus. The most commonly mentioned include the requirements of the replication machinery and interactions with the cellular partners, RNA structure stability, and the immune responses triggered at higher temperatures (74, 75). For instance, in a study on human rhinovirus, it was found that type I and type III interferon-mediated responses are inefficient at lower temperatures of the airway tract, which contributed to more productive viral replication at this site (76, 77). The observed universal permissivity at 32 °C in this study also aligns with the findings of Foxman et al. (76), who established that lower temperatures (characteristic of the upper respiratory tract) diminish the potency of the innate antiviral defense. Our data suggest that HCoV-229E may similarly benefit from a thermal threshold for innate immune sensing; at 32 °C, the lack of robust ISG induction across all donors indicates a window of “immune ignorance” that facilitates viral replication. Consistent with viral intrinsic temperature sensitivity, previous work in human airway organoids demonstrated markedly higher replication of HCoV-229E at 33 than 37 °C, reflecting a preference for a cooler upper-respiratory-tract temperature (10). Classical infectivity studies also indicate that HCoV-229E stability and infectivity decrease at higher temperature (13, 78). In our study, the persistence of replication in fetal and pediatric enteroids at 37 °C may reflect developmental differences, including transcriptional programs supporting translation, ribosome biogenesis, and attenuated innate immune responses, which can compensate for thermal constraints. In contrast, aHIEs likely restrict replication at 37 °C due to less permissive host machinery and enhanced antiviral responses. To date, HIEs studies have largely focused on SARS-CoV and SARS-CoV-2 (51, 52, 67), without evaluating viral fitness across different temperatures. Moreover, although enteric coronavirus models exist in nonhuman enteroids [e.g., PEDV (79), equine coronavirus (80), or FECV (81)], these have not explored the temperature-dependent effect either. Thus, our finding that HCoV-229E replication is robust at 32 °C across all donor enteroids but restricted in aHIEs at 37 °C, fills a critical knowledge gap. It suggests that the immature intestinal epithelium provides a unique thermal niche influencing viral permissiveness and potentially shaping age-dependent susceptibility to seasonal HCoVs.

Additionally, at 37 °C, we also observed upregulation of metabolic and homeostatic pathways consistent with elevated energetic and membrane demands, including hypoxia-responsive pathways. Lipid metabolism and energy production pathways were prominently upregulated, reflecting a shift toward a stress-adaptive epithelial state. This temperature-induced response was strongly age-dependent: aHIEs failed to induce many stress-response and cell–cell adhesion genes observed in fetal and pediatric tissues, indicating diminished transcriptional plasticity in a mature epithelium. Together, reduced translation and enhanced metabolic adaptation at 37 °C point to a reciprocal regulatory axis that may directly influence viral replication. These findings are in line with the described developmental differences among HIEs. Infant and adult enteroids’ gene expression profiles differ substantially, especially in genes involved in transcriptional identity, maturation, gut barrier function, metabolic features, and innate immunity (44, 82). Fetal HIEs maintain early developmental signatures, including differential expression of adhesion, proliferation, and immune pathways, which may contribute to their heightened viral permissiveness (82).

To define the HCoV-229E cellular tropism, we mapped the permissive cell populations within the intestinal epithelium. We observed that enterocytes make up the majority of intestinal cells targeted by HCoV-229E, similarly as reported for SARS-CoV, SARS-CoV-2, and PEDV in porcine intestinal epithelia (51, 66, 79). In contrast to SARS-CoV-2, which exclusively infects enterocyte-lineage cells (51), we found that HCoV-229E also infected Paneth cells, goblet cells, and enteroendocrine cells but only at 32 °C. Within the human intestinal epithelium, ANPEP expression is primarily localized to mature enterocytes, with minimal to negligible detection in stem, progenitor, or other secretory cell lineages (83). Given the abundance of ANPEP in the small intestine and the observation that HCoV-229E can use TMPRSS2-dependent entry at the cell surface in primary tissues (61, 84), these findings suggest that tropism is not restricted to a single epithelial lineage. Consistent with this, camostat, a serine protease inhibitor, markedly suppressed infection, highlighting a dependence on protease-mediated entry.

Although we showed that HCoV-229E can infect and replicate within the intestinal epithelium, the route by which seasonal HCoVs disseminate beyond the respiratory tract remains unclear (32, 85, 86). One may not, however, reject the gastrointestinal route. While digestive juices and low pH inactivate HCoV-229E under fed-state conditions (26), mucus aggregates have been shown to protect respiratory viruses and facilitate delivery to distal sites (87). Furthermore, HCoV-229E can infect and persist in human monocytes, enhancing migration and suggesting a potential mechanism for systemic dissemination via infected immune cells (88). Some other limitations of our study should be recognized. First, while HIEs recapitulate many features of intestinal epithelium, they lack components of the in vivo mucosal environment, such as immune cells, neural or stromal components, microbiota, or nutrient gradients along the crypt–villus axis. These factors might further modulate susceptibility in ways not captured by our model. Second, the number of donors, particularly in pediatric and adult groups, may constrain generalizability, and interdonor variability that contributes to the observed differences. Third, the available HKU1 and OC43 inocula had substantially lower viral titers than those of 229E and NL63, and we were unable to generate high-titer stocks. Hence, one cannot overlook the possibility that these viruses might replicate in HIEs if a higher amount of starting inoculum is administered. Finally, given that our study evaluated only one strain from each seasonal coronavirus, the extent to which variant-to-variant differences may alter tropism or host dependencies remains an open question.

In conclusion, our study demonstrates that age-, temperature-, and donor-dependent epithelial states critically shape susceptibility to HCoV-229E intestinal infection. By establishing age-stratified HIEs as a model for coronavirus–epithelium interactions, we provide a foundation for a deeper dissection of how developmental stages and physiological niches influence viral permissiveness and tropism and highlight host pathways that may be particularly relevant for viral pathogenesis and therapeutic intervention.

## Materials and Methods

### Human Enteroids: Patient Sample Collection and Enteroid Isolation.

Adult aHIEs derived from the ileum were purchased from Sigma Aldrich (Prep ht-105-I, Product #: SCC339 and Prep ht-104-I, Product #: SCC337). Pediatric enteroid lines (pHIEs) T363 and T354 were a kind gift from Dr. Matthias Zilbauer (Department of Pediatrics, University of Cambridge, United Kingdom), derived from the duodenum of a healthy, 6-y-old donor and a healthy, 7-y-old donor, respectively. The duodenal biopsies for the T363 and T354 enteroids were harvested from a study conducted with informed patient and/or carer consent as appropriate and with full ethical approval (REC-17/EE/0265). Fetal intestinal tissues from donors were obtained by the Amsterdam University Medical Center from a private clinic. The tissues were used to derive fetal HIEs (fHIEs) #210087 and #210088. The donors for these enteroids were respectively 16 and 19 wk old. To ensure privacy, the clinic retains all written informed consent forms, and the data provided to Amsterdam UMC is anonymized, precluding donor identification. This research adheres to Dutch legal standards governing the use of such biological materials. For cases 1112 and 1136 (fetal), small intestinal tissues were collected from fetal elective terminations without congenital anomalies at the University of Pittsburgh with Institutional Review Board approval and informed consent. Samples were cryopreserved as previously described (89). These samples were maintained in the Konnikova Lab Biorepository. All HIEs used in this study are summarized in *SI Appendix*, Table S1. HIEs from all donors were derived from healthy, nontransformed tissue sections.

Isolated adult and pediatric intestinal crypts were seeded in Cultrex UltiMatrix Basement Membrane Extract with a reduced growth factor (R&D systemsSystems, BioTechne), whereas fetal intestinal crypts were seeded in Corning® Matrigel® Matrix growth factor-reduced, Phenol Red-free, and LDEV-Free (Corning #356231, USA). All seeded intestinal crypts were maintained in Human IntestiCult™ Organoid Growth Medium (OGMh, STEMCELL™ Technologies #06010) supplemented with 100 units/mL penicillin and 100 µg/mL streptomycin (1% P/S, Gibco) and 10 µM Y-27632 (Sigma-Aldrich), referred in this manuscript as proliferation medium (PM). Enteroids were incubated at 37 °C in an atmosphere containing 5% CO_2_ unless otherwise stated. All enteroids were derived from healthy, nontransformed tissue sections of each respective donor.

### HIEs Passaging and Maintenance.

HIEs were passaged according to general recommendations from StemCell. Briefly, media were removed from each well and saved for routine mycoplasma testing (HIEs’ supernatants were tested for mycoplasma contamination every 2 wk). For expansion, enteroids cultured in domes were harvested, and single-cell suspensions were obtained by treatment with TrypLE™ (Gibco, Thermo Fisher Scientific, Poland) for 10 to 15 min at 37 °C, followed by inactivation in Advanced DMEM/F12 supplemented with 1% P/S, 10 mM HEPES (Gibco, Thermo Fisher Scientific, Poland), and 1× GlutaMAX™ (Gibco, Thermo Fisher Scientific, Poland) (referred to as AdDMEM+++), additionally supplemented with 15% FBS to inactivate trypsin. Cells were then centrifuged at 500×*g*, 4 °C for 5 min and resuspended in Matrigel. Organoids were passaged at a ratio of approximately 1:2 or 1:3 and plated in 25 µL Matrigel/dome with ~10 domes per one well (6-well plate). After basement membrane polymerization at 37 °C/5% CO_2_, 1.5 mL PM/well was added. HIEs were maintained at 37 °C/5% CO_2_ for passage every 6 to 10 d with PM replacement every 48 to 72 h.

### Human Enteroid Monolayer Culture.

Enteroid monolayers were established as previously described (40, 90–92). Briefly, Transwell^®^ cell culture inserts (6.5 mm, 0.4 µm pore size, VWR) were coated with 100 µL of 20 µg/mL rat tail collagen type 1 (Ibidi, Germany) in 0.1% (v/v) acetic acid for at least 1 h at 37 °C and washed twice with 1× PBS before seeding. Enteroid domes were initially harvested, and single-cell suspensions were obtained by treatment with TrypLE™ (Gibco, Thermo Fisher Scientific, Poland) for 10 min at 37 °C, followed by inactivation in AdDMEM+++, additionally supplemented with 15% FBS to inactivate trypsin. Cells were then centrifuged at 500×*g*, 4 °C for 5 min and resuspended in prewarmed PM. ~1 × 10^5^ cells were seeded in 100 µL of the medium onto each insert, and 300 µL of PM was added to the basolateral site. The PM was refreshed every 3 to 4 d until monolayer was confluent. Once the monolayer was confluent, PM was replaced with a differentiation medium (DM) consisting of a 1:1 (v/v) mix of OGMh human basal medium component and AdDMEM+++, to stimulate cell differentiation. The DM was refreshed every 3 to 4 d, and monolayers were maintained for 7 d.

### Viruses.

HCoV-229E was isolated from an anonymous patient at Amsterdam University Medical Center on March 20, 2017, and was kindly gifted by Martin Deijs and Dr. Lia van der Hoek (both from the Laboratory of Experimental Virology, Amsterdam University Medical Center, The Netherlands). The procured isolate of HCoV-229E was propagated once in human airway epithelia (HAE), followed by another round of propagation in Huh-7 cells (56). A low-passage virus was used in this study. Infectious viral titers were determined according to the Reed & Muench method (93) by infecting confluent A549^++^ cells on 96-well plates with 10-fold serial dilutions of viral stocks or supernatants prepared in DMEM + 1% P/S, and the readout was carried out on day 6 p.i. The titer of HCoV-229E stocks used in this study was 8.69 × 10^6^ TCID_50_/mL. The HCoV-229E used in this study was genotyped using NCBI BLAST and was found to have 99.89% genomic sequence identity with HCoV-229E strain Seattle/USA/SC2872/2015 (GenBank ID: KY967357.1) (56). HCoV-HKU1 strain Caen 1 was obtained from a clinical specimen collected in March 2005 at the pediatric department of the University Hospital of Caen, France (94). HCoV-OC43 isolate 0500 was kindly gifted by Prof. Volker Thiel (Institute of Virology and Immunology, Switzerland) and propagated in fully differentiated HAE (95). HCoV-NL63 isolate Amsterdam 1 clinical strain was propagated in HAE. All virus stocks were aliquoted and stored at −80 °C.

### Infection of Enteroid Monolayers.

For the initial exploratory infection of enteroids with seasonal, low-pathogenic HCoVs, confluent enteroid monolayers were inoculated apically with 50 µL of 10× diluted stock of HCoV-229E clinical isolate, HCoV-NL63 isolate Amsterdam 1, HCoV-OC43 isolate 0500, or HCoV-HKU1 isolate Caen 1 following removal of DM. After an initial 2 h incubation at 32 °C, the unbound virus was removed by washing the wells twice with prewarmed AdDMEM+++. Subsequently, 100 µL and 600 µL of fresh, prewarmed DM were reapplied to the apical and basolateral compartments, respectively. Enteroid monolayers were then incubated at 32 °C. The apical culture medium (20 µL) was collected at 2 h p.i. and then every 24 h. The sampled volume was replaced with a fresh, prewarmed DM each time.

For subsequent experiments with HCoV-229E clinical isolate, confluent enteroid monolayers were similarly inoculated apically with the virus at 1.43 × 10^4^ or 1.43 × 10^5^ TCID_50_/ml each unless otherwise stated. After an initial 2 h incubation at either 32 or 37 °C, unbound virus was removed by washing the wells twice with prewarmed AdDMEM+++. After replenishment of DM at the apical and basolateral sides as described above, enteroid monolayers were incubated at either 32 or 37 °C. The apical culture medium (20 µL) was collected at 2, 24, 48, 96, and 120 h p.i. The sampled volume was replaced with a fresh, prewarmed DM each time.

### Statistical Analyses.

Statistical calculations were performed using the GraphPad Prism 9 software and R (version 4.3.3) with default options. Data are presented as the mean ± SD or mean SEM for parametric datasets and median ± IQR (interquartile range) for nonparametric datasets. The normality of data was evaluated using the Shapiro–Wilk test, and the F-test was used to test the assumption of equality of variances. A value of *P* < 0.05 was considered significant for all analyses.

The TEER measurement, cell lines, RNA extraction, RT-qPCR, subgenomic RNA detection, bulk RNA sequencing (RNA-seq), Data analysis of RNA sequencing (RNA-seq) and DEG analysis, Gene ontology (GO) overrepresentation analysis, Immunofluorescence Assay, Flow Cytometry, and viral entry inhibition assay detailed methods are described in *SI Appendix*.

## Supplementary Material

Appendix 01 (PDF)

Dataset S01 (XLSX)

Dataset S02 (XLSX)

Dataset S03 (XLSX)

Dataset S04 (XLSX)

## Data Availability

All RNA sequencing data in this study have been deposited in the Gene Expression Omnibus (GEO) database under Accession No. GSE320363 (96). All other data are included in the manuscript and/or supporting information.
