# Supplementary material for: Temperature and developmental stage govern intestinal susceptibility to human coronavirus 229E
Source: Proc Natl Acad Sci U S A. 2026 Jun 24;123(26):e2600632123. doi: 10.1073/pnas.2600632123 (PMC13320717; doi:10.1073/pnas.2600632123)
Supplement: Supplementary file 1 — Appendix 01 (PDF) [file pnas.2600632123.sapp.pdf]

PNAS

Supporting Information for

Temperature and developmental stage govern intestinal susceptibility to human coronavirus 229E.

Aleksandra Synowiec [a,b,c,d,#,*], Laurensius Kevin Lie [a,#], Katarzyna Owczarek [a], Nina Johannesson [e,f], Nina Mickiewicz [a,b,g], Heng-Chang Chen [h], Artur Szczepański [a], Madison S. Strine [d], Renata B. Filler [c], Maciej Borowiec [i], Michał Pietrusiński [i], Izabela Dróżdż [i], Agnieszka Robaszkiewicz [j], Michał Bochenek [a], Matthias Zilbauer [k], Justyna Rymarowicz [l], Michał Pędziwiatr [l], Liza Konnikova [d], Craig B. Wilen [c,d], Dasja Pajkrt [e], Katja C. Wolthers [f], Carlemi Calitz [e,f], Adithya Sridhar [e,f,m], Krzysztof Pyrc [a,*].

(a) Malopolska Centre of Biotechnology, Jagiellonian University, Krakow, Poland
(b) Doctoral School of Exact and Natural Sciences, Jagiellonian University, Krakow, Poland
(c) Department of Laboratory Medicine, Yale School of Medicine, New Haven, CT, USA
(d) Department of Immunobiology, Yale School of Medicine, New Haven, CT, USA
(e) OrganoVIR Labs, Emma Children's Hospital, Department of Pediatric Infectious Diseases, Amsterdam UMC, Academic Medical Center, Amsterdam Institute for Infection and Immunity, Amsterdam Institute for Reproduction and Development, University of Amsterdam, Amsterdam, The Netherlands
(f) OrganoVIR Labs, Department of Medical Microbiology, Amsterdam UMC, Academic Medical Center, Amsterdam Institute for Infection and Immunity, University of Amsterdam, Amsterdam, The Netherlands
(g) Microbiology Department, Faculty of Biochemistry, Biophysics and Biotechnology, Jagiellonian University, Krakow, Poland.
(h) Quantitative Virology Research Group, Population Diagnostics Center, Łukasiewicz Research Network – PORT Polish Center for Technology Development, Wrocław, Poland
(i) Department of Clinical and Laboratory Genetics, Department of Clinical Genetics, Medical University of Lodz, Poland
(j) Department of General Biophysics, Faculty of Biology and Environmental Protection, University of Lodz, Poland
(k) Department of Pediatrics, School of Clinical Medicine, University of Cambridge, United Kingdom
(l) Department of General Surgery, Jagiellonian University Medical College, Krakow, Poland
(m) Emma Center for Personalized Medicine, Amsterdam UMC, Amsterdam, the Netherlands

(#) First co-authors
(*) Corresponding authors

Correspondence should be addressed to Krzysztof Pyrc (k.a.pyrc@uj.edu.pl) or Aleksandra Synowiec (aleksandra.synowiec@uj.edu.pl);
www: http://virogenetics.info/

This PDF file includes:

Supplementary Methods:

- Transepithelial electrical resistance (TEER)
- Cell lines
- RNA extraction
- RT-qPCR
- Subgenomic RNA detection
- Bulk RNA Sequencing (RNA-Seq)
- Data analysis of RNA sequencing (RNA-seq) and differential gene expression analysis
- Gene ontology (GO) over-representation analysis.
- Immunofluorescence assay
- Flow cytometry
- Viral entry inhibition assay

Supplementary Figures S1 to S7

Supplementary Tables S1 to S3

Legends for Datasets S1 to S4

Supplementary Methods

Transepithelial Electrical Resistance (TEER) measurement

To assess enteroid monolayer integrity, transepithelial electrical resistance (TEER) measurement of enteroid monolayers was carried out as previously described using EVOM2 Epithelial Volt/Ohm Meter (WPI)[2-5]. Briefly, the culture medium was refreshed 30 minutes before the measurement, and each enteroid culture well was evaluated as follows: mean values of three measurements were corrected for background TEER and surface area of the insert to obtain net-area resistance in Ω/cm^2. Enteroid monolayers with TEER values above 200 Ω/cm^2 were used for further assays (***Supplementary Figure S1***).

Cell lines

A549 cells overexpressing CD13 and TMPRSS2, henceforth referred to as A549^{++} cells, have been generated in-house[6]. A549^{++} cells were maintained in Dulbecco's minimum essential medium 1× (DMEM, Gibco) supplemented with 5% Fetal Bovine Serum (5% FBS, Gibco), 1% P/S, 10 µg/ml of blasticidin S (Thermo Fisher Scientific), and 500 µg/ml of G418 (Geneticin™, Sigma-Aldrich). A549^{++} cells were passaged every 3 to 4 days and maintained at 37°C and 5% CO_2 unless otherwise stated. All enteroids and cultured cells were tested every two weeks for mycoplasma contamination either by 4',6-diamidino-2-phenylindole (DAPI) staining
or LookOut® Mycoplasma PCR Detection Kit (Sigma-Aldrich).

RNA extraction

RNA was isolated from 20 µl of culture medium sample taken from enteroid monolayers using the MagnifiQ™ viral RNA kit (A&A Biotechnology) on the KingFisher™ Flex Purification System with 96 PCR head platform (Thermo Fisher Scientific), according to the respective manufacturers' protocol. For RNA-seq analysis, RNA was extracted using Direct-zol RNA Miniprep Plus (Zymo Research).

RT-qPCR

To quantify the nucleic acids, 3 µl of the eluted RNA were used for one-step RT-qPCR using the GoTaq® Probe one-step RT-qPCR system (Promega) according to the manufacturer's protocol in the presence of the primers and probes presented in ***Supplementary Table S2***. The reaction was carried out using a CFX96™ Touch Real-Time PCR Detection System (Bio-Rad Laboratories). C_q values were transformed into viral RNA copies using a standard curve with a known number of viral genome copies. The reaction was performed as follows: 15 minutes at 45°C and 2 minutes at 95°C, followed by 40 cycles of 15 seconds at 95°C and 1-minute elongation at 60°C for all HCoVs.

Subgenomic RNA detection

Lysates of HCoV-229E-infected enteroid monolayers were collected for whole RNA extraction at 48 h p.i. and 96 h p.i. Eluted RNA was processed using a TURBO™ DNAse kit (Invitrogen) according to the manufacturer's protocol to remove genomic DNA. RNA was reverse-transcribed, and an equal amount of the complementary DNA (cDNA) was used as a template for a subgenomic mRNA (sg mRNA)-specific PCR, comprised of an initial conventional PCR followed by a semi-nested PCR. The sg mRNA-specific PCR was performed using 1× Dream *Taq* Green PCR Master Mix (Thermo Fisher Scientific, Poland) and appropriate primers (***Supplementary Table S2***). A primer set spanning the complementary DNA sequence corresponding to the putative leader-body junction of the nucleocapsid (*N*) sgRNA has been previously described[7,8]. For the initial PCR, leader-specific sense (25-up leader) and antisense primer for the first PCR (25900-down) were used. For the semi-nested PCR, the 25-up leader primer and the antisense primer for the nested PCR (25820-down) were used. *GAPDH* mRNA was used as a housekeeping gene reference. The reaction was performed as follows: 3 minutes at 95°C, 35 cycles (30 for nested PCR) of (30 seconds at 95°C, 30 seconds at 53°C, and 20 seconds at 72°C), followed by 5 minutes at 72°C and 10 minutes at 4°C. The entire volume of each nested PCR product was run on 2.5% agarose gel at 100V for 40 minutes, and resultant DNA bands were imaged using ChemiDoc™ MP Imaging System (Bio-Rad Laboratories).

Bulk RNA Sequencing (RNA-Seq)

RNA sequencing libraries were prepared using the QIAseq Stranded RNA Library Kit (Qiagen, Germany) following the manufacturer's protocol, with integrated globin transcript depletion using the QIAseq FastSelect – Globin Kit (Qiagen, Germany). A total of 1 µg of high-quality total RNA per sample (RNA integrity number ≥ 7) was used as input. RNA concentration was quantified fluorometrically using the Qubit RNA High-Sensitivity Assay Kit (Thermo Fisher Scientific), and integrity was assessed using an Agilent 2100 Bioanalyzer (Agilent Technologies). Globin transcript depletion was performed directly within the first-strand synthesis step according to Qiagen's recommended hybridization protocol. For each reaction, 1 µg of total RNA was mixed with 1 µl of FastSelect – Globin oligonucleotide reagent, 8 µl of 5× Reverse Transcription (RT) Buffer, 2 µl of RT Enzyme Mix, and nuclease-free water to a final volume of 37 µl. The mixture was transferred to a thermal cycler and subjected to a stepwise temperature reduction to promote oligonucleotide hybridization to globin transcripts and fragmentation of RNA. The thermal program consisted of 95°C for 3 min, followed by successive 2-minute incubations at 75°C, 70°C, 65°C, 60°C, 55°C, 50°C, 37°C, and 25°C, with a final hold at 4°C. Immediately following the hybridization step, first-strand cDNA synthesis was carried out in the same tube. Reverse transcription was performed using

the kit's RT enzyme mix (1 µl of 0,4M DTT; 1 µl of RT Enzyme; 1 µl of RNase Inhibitor) under the following conditions: 25°C for 10 min to allow primer annealing, 42°C for 15 min for cDNA extension, and 70°C for 15 min to terminate the reaction. The resulting first-strand cDNA was purified using QIAseq Beads (Qiagen) at a bead-to-sample ratio of 1.8×. The bound cDNA was washed twice with 80% ethanol, air-dried, and eluted in 38,5 µl of nuclease-free water. Double-stranded cDNA was generated using the Second Strand Synthesis Buffer and Enzyme Mix provided with the kit. The purified first-strand cDNA (38,5 µl) was combined with 5 µl of 10× Second Strand Buffer, 6.5 µl of Enzyme Mix to reach a total reaction volume of 50 µl. The reaction was incubated at 25°C for 30 min and then 65°C for 15 min to allow second-strand synthesis and simultaneous end-repair and A-tailing. Following incubation, the product was purified again using 1.8× QIAseq Beads and eluted in 50 µl of nuclease-free water. To enable sequencing on Illumina platforms, adapters containing unique dual indices were ligated to the A-tailed cDNA fragments. Each 50 µl of purified cDNA was combined with 2 µl of Adapter Mix and 48 µl of Ligation Master Mix (total volume 100 µl). The ligation reaction was incubated at 25°C for 10 min. After which, adapter-ligated products were purified using QIAseq Beads at a 0.9× ratio to remove unligated adapters and adapter dimers. The final ligated library DNA was eluted in 23,5 µl of nuclease-free water. Adapter-ligated cDNA fragments were amplified using the HiFi PCR Master Mix provided in the kit. Each 50 µl PCR reaction contained 23,5 µl of ligated DNA, 25 µl of HiFi Master Mix, and 1,5 µl of Primer Mix. PCR was performed on a thermal cycler under the following conditions: initial denaturation at 98°C for 2 min; 13 cycles of 98°C for 20 s, 60°C for 30 s, and 72°C for 30 s; final extension at 72°C for 1 min; and a hold at 4°C. Amplified libraries were purified using a 1.0× bead clean-up to remove primer dimers and small fragments, and the final products were eluted in 20 µl of nuclease-free water. nLibrary quality and fragment size distribution were evaluated using a TapeStation HS D1000 ScreenTape. Typical library size distributions ranged from 300 to 500 bp for the absence of adapters or adapter-dimers (130 bp). Libraries were quantified fluorometrically using the Qubit dsDNA High Sensitivity Assay (Thermo Fisher Scientific) and by qPCR using the Qiaseq Library Quant Array Kit (Qiagen, Germany). Libraries showing low adapter-dimer content (< 5%) and sufficient yield (> 10 nM) were selected for sequencing. Indexed libraries were normalized to 4 nM, pooled equimolarly, and denatured following Salus BioMed standard denaturation protocol. Sequencing was performed on a Salus PRO platform (Salus BioMed, Shenzhen, CRL) to generate paired-end 2 × 150 bp reads. Strandedness was preserved according to the QIAseq chemistry, such that Read 1 corresponds to the sense strand of the original RNA molecule.

Data analysis of RNA sequencing (RNA-seq) and differential gene expression analysis

Sequencing reads from total RNA isolated from 48 samples from six donors across three age categories (fetal, pediatric, and adult) and two temperatures (32ºC and 37ºC) were mapped to GENCODE protein-coding transcript sequence Human GRCh38.p14, released 45[9,10] with kallisto[11] with options 'quant' (the quantification algorithm), '–single' (single-end mode), '–s 20' (fragment length 20 nucleotides), and '–l 200' (s.d. 200 nucleotides). The counts of the different isoforms were summed, thus generating a total count per gene copy in transcripts per million. The output file of the quantified transcript abundances was used for differential gene expression analysis. Differential gene expression analysis was performed in R using the R packages "tximport"[12] and "DESeq2"[13]. The index table was built using TxDb.Hsapiens.UCSC.hg38.knownGene (Team BC and Maintainer BP, 2019). Two experimental settings were set for this analysis. In the mock setting, analysis was performed by comparing two temperatures (32°C versus 37°C). Two biological replicates in each condition (32°C versus 37°C) were applied to each donor; the analysis was conducted independently for each donor. In the HCoV-229E-infected setting, two variables (age categories and infection) were taken into account for the pairwise comparison. The analysis was conducted independently for temperature.

Files of transcript abundances quantified by kallisto[11] were imported using the function tximport() followed by the function DESeqDataSetFromTximport() to construct a DESeqDataSet from. The result table was extracted using the function result() from the R package "DESeq"[14]. In the former setting, the condition treated at 32°C serves as a reference control; in the latter setting, the age category and pediatric serve as a set of reference controls. The genes with adjusted *p*-values smaller than 0.05 were retrieved for those with differential gene expression. Among them, the genes with a fold change greater than 0.6 on a logarithmic scale were designated as up-regulation, whereas the genes with a fold change smaller than -0.6 on a logarithmic scale were designated as down-regulation. The HCoV-229E reference genome[6], and corresponding sg mRNA sequences were used to generate kallisto[11] index files, allowing computing a total count per viral gene copy in transcripts per million. The nucleotide "U" was converted to "T" before indexing the file with the command line described below.

```
> awk '/^[^>]/{ gsub(/U/,"T") }1' 229E_CoV_KAP.genome.fa >
229E_CoV_KAP.genome.convert.fa
```

Sequencing reads from total RNA isolated from 24 samples subjected to HCoV-229E infection from six donors across three age categories (fetal, pediatric, and adult) were independently mapped to mentioned reference HCoV-229E genome and corresponding sg mRNA sequences with kallisto[11] with options 'quant' (the quantification algorithm), '–single' (single-end mode),

'–s 20' (fragment length 20 nucleotides), and '–l 200' (s.d. 200 nucleotides). All analytical codes are available at GitHub (https://github.com/HCAngelC/HCoV-229E.RNA.seq/).

Gene ontology (GO) over-representation analysis.

The analysis was performed using the function enrichGO() in the R package clusterProfiler (Version 4.4.1)[15,16] with the subontology, biological processes (BP), and the cutoff of the adjusted *p*-value and the *q*-value equal to 0.05 based on the genes with differential gene expression. Analyses were independently performed using differentially expressed genes, separated between up- and down-regulation across six donors (***Supplementary Table S1***). Rich factors representing the magnitude of over-representation of the identified gene sets were calculated by dividing GeneRatio by BgRatio with the command lines described below[16,17], as shown in bubble plots.

```
> GO_output_file$GeneRatio <-as. numeric(gsub("(\\d+)/(\\d+)", "\\1",
GO_output_file$GeneRatio, perl = T))/as.numeric(gsub("(\\d+)/(\\d+)", "\\2",
GO_output_file$GeneRatio, perl = T)) # Convert GeneRatio to numerical variables.

> GO_output_file$BgRatio <- as.numeric(gsub("(\\d+)/(\\d+)", "\\1", GO_output_file$BgRatio,
perl = T))/as.numeric(gsub("(\\d+)/(\\d+)", "\\2", GO_output_file$BgRatio, perl = T))# Convert
BgRatio to numerical variables.

> GO_output_file <- GO_output_file %>% dplyr::mutate(rich_factor = GeneRatio/BgRatio)#
Calculate rich factors.
```

Immunofluorescence Assay

Enteroid monolayers in Transwell™ inserts were washed twice with 1× PBS and fixed in 4% paraformaldehyde (Sigma-Aldrich, Poland) for 20 minutes at RT. Membranes were washed three times with 1× PBS before excision from the insert, followed by permeabilization in permeabilization buffer (0.5% TRITON-X (Bioshop, Canada) in 1×PBS) for 15 min at RT. Cells were then washed twice with 1× PBS and incubated overnight at 4°C in blocking buffer (10% BSA in 1× PBS). The next day, blocking buffer was removed and cells were incubated for 2h at room temperature (RT) with primary antibodies diluted in 2.5% BSA containing 0.1% Tween-20 (Bioshop, Canada) in 1× PBS. Cells were then washed twice with washing buffer (0.1% Tween-20 in 1× PBS) and incubated with secondary antibodies diluted in 2.5% BSA with 0.5% Tween-20 for 1 h at RT. The exact dilutions of primary and secondary antibodies are listed in ***Supplementary Table S3***. Afterward, monolayers were washed three times in 1×PBS and incubated for 15 min at RT with 0.1 µg/ml of DAPI (Sigma-Aldrich, Poland). Monolayers were then rewashed thrice in 1× PBS and mounted on glass slides in ProLong™ Diamond Antifade Mountant (Invitrogen). Images were acquired under a Zeiss LSM 710 confocal microscope (Carl Zeiss Microscopy GmbH; release version 8.1) using ZEN 2012 SP1 software

(Carl Zeiss Microscopy GmbH; black edition, version 8.1.0.484). Images were processed using the ImageJ FIJI version 2.9.0 (National Institutes of Health, Bethesda, MD, USA)[18] and are presented as the maximal projections unless stated otherwise.

Flow Cytometry

Enteroid monolayers cultured in Transwell™ inserts were washed twice with 1× PBS and dissociated using 100 µl per insert of TrypLE Express Enzyme (Gibco, Thermo Fisher Scientific, Poland) for 15–20 min at 37°C. After detachment, 100 µl of AdMEM+++ supplemented with 15% FBS was added, and cells were transferred to 1.5 ml tubes and centrifuged at 300 × g for 5 min at 4°C. Pelleted cells were washed twice with 1× PBS under the same centrifugation conditions and fixed in 4% paraformaldehyde (in 1× PBS) for 20 min at room temperature (RT). Following fixation, cells were washed by centrifugation at 500 × g for 5 min at 4°C (all subsequent washes were performed similarly) and permeabilized with permeabilization buffer (0.05% TRITON X-100 in 1× PBS) for 15 min at RT with gentle rocking. Cells were then washed twice with 1× PBS and incubated overnight at 4°C in blocking buffer (10% BSA in 1× PBS) with rocking. The next day, blocking buffer was removed, and cells were incubated overnight at 4°C with primary antibodies diluted in 1% BSA containing 0.1% Tween-20 (Bioshop, Canada) in 1× PBS, with rocking. Cells were then washed twice with washing buffer (0.1% Tween-20 in 1× PBS) and incubated with secondary antibodies diluted in 1% BSA with 0.5% Tween-20 for 1 h at room temperature with rocking. The exact dilutions of primary and secondary antibodies are listed in ***Supplementary Table S3***. Following staining, cells were washed twice with washing buffer, followed by two final washes with 1× PBS, and analyzed on a Navios flow cytometer (Beckman Coulter).

Viral entry inhibition assay

The following inhibitors were used in this study: camostat mesylate (10 µM working concentration, Sigma Aldrich, Poland), E64d (10 µM working concentration, Sigma Aldrich, Poland), ammonium chloride (NH_4Cl, 10 mM working concentration, Sigma-Aldrich, Poland), and bafilomycin A1 (10 nM working concentration, Sigma Aldrich, Poland). Initially, basolateral and apical media of confluent, differentiated enteroid monolayers were replaced with DM containing respective inhibitors at 2× working concentration, with 50 µl on the apical side and 300 µl at the basolateral side. Enteroid monolayers were then preincubated at 32°C for 1 h. Following preincubation, media at both sides were replaced with DM containing respective inhibitors at 1× working concentration, and additionally HCoV-229E clinical isolate at 1.43 × 10^4 $TCID_{50}$/ml was added onto the apical side. Monolayers were incubated for 2 h at 32°C, followed by inoculum removal and two washes of the apical side with pre-warmed AdDMEM+++. After that, DM with or without an inhibitor was added to both sides (100 µl apical side and 300 µl basolateral side) and monolayers were further incubated at 32°C for 48 h.

Finally, 20 µl of apical culture medium was extracted at 2 h, 24 h, and 48 h p.i. to quantify viral RNA by RT-qPCR. At the 48 h p.i. monolayers were fixed with 4% paraformaldehyde and subjected to an immunostaining assay.

Supplementary Data

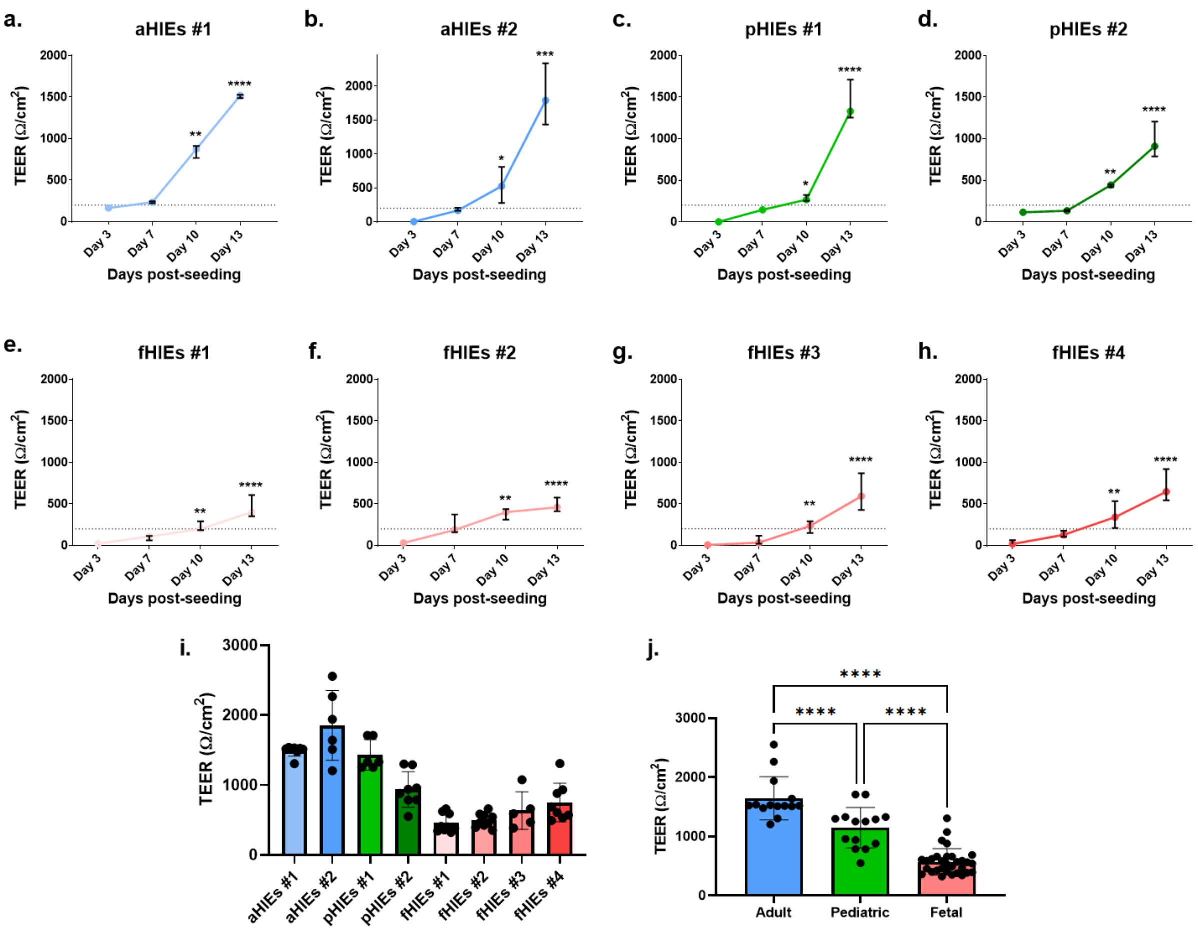

Supplementary Figure S1. Transepithelial electrical resistance (TEER) comparison among fetal, pediatric, and adult HIEs. TEER measurements were used to assess the differentiation status and barrier integrity of HIEs derived from adult (**a**, **b**), pediatric (**c**, **d**), and fetal (**e**, **f**, **g**, **h**) donors. (**i**) TEER was measured on Day 13 of differentiation in monolayers derived from all donors. (**j**) TEER values from individual donors were pooled within each age group and compared across groups. Data are acquired from at least two independent experiments performed in triplicate and presented as median ± IQR (**a-i**) or mean ± SEM (**j**). Statistical analyses were performed using the Friedman test with Dunn's post hoc correction (**a-h**) or one-way ANOVA with Tukey post hoc correction (**j**). * P <0.05, ** P< 0.01, *** P < 0.001, **** P < 0.0001.

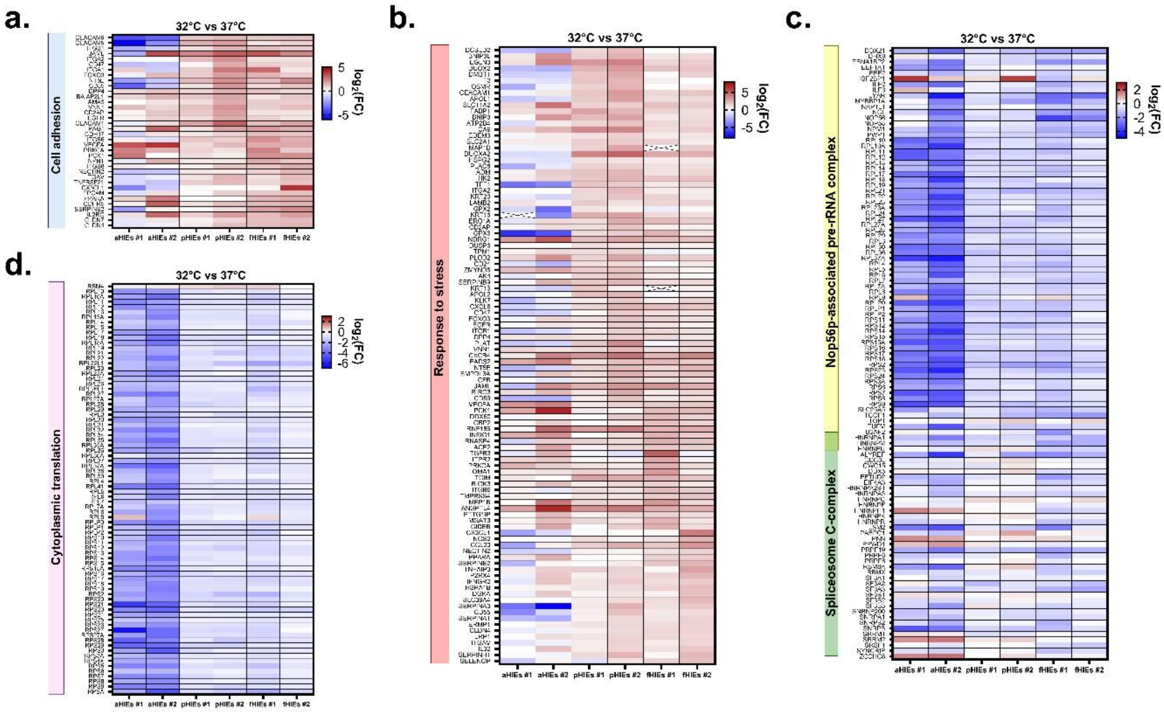

Supplementary Figure S2. Temperature-dependent change of transcriptional signatures in HIEs across donors' age. (a–d) Heatmaps displaying log_2FC (fold change) (32°C vs. 37°C) for genes associated with (**a**) cell adhesion, (**b**) Response to stress, (**c**) Spliceosome C-complex and Nop56p-associated pre-rRNA complex, and (**d**) cytoplasmic translation. "X" indicates genes excluded from differential expression analysis due to low or zero counts, resulting in no reported value.

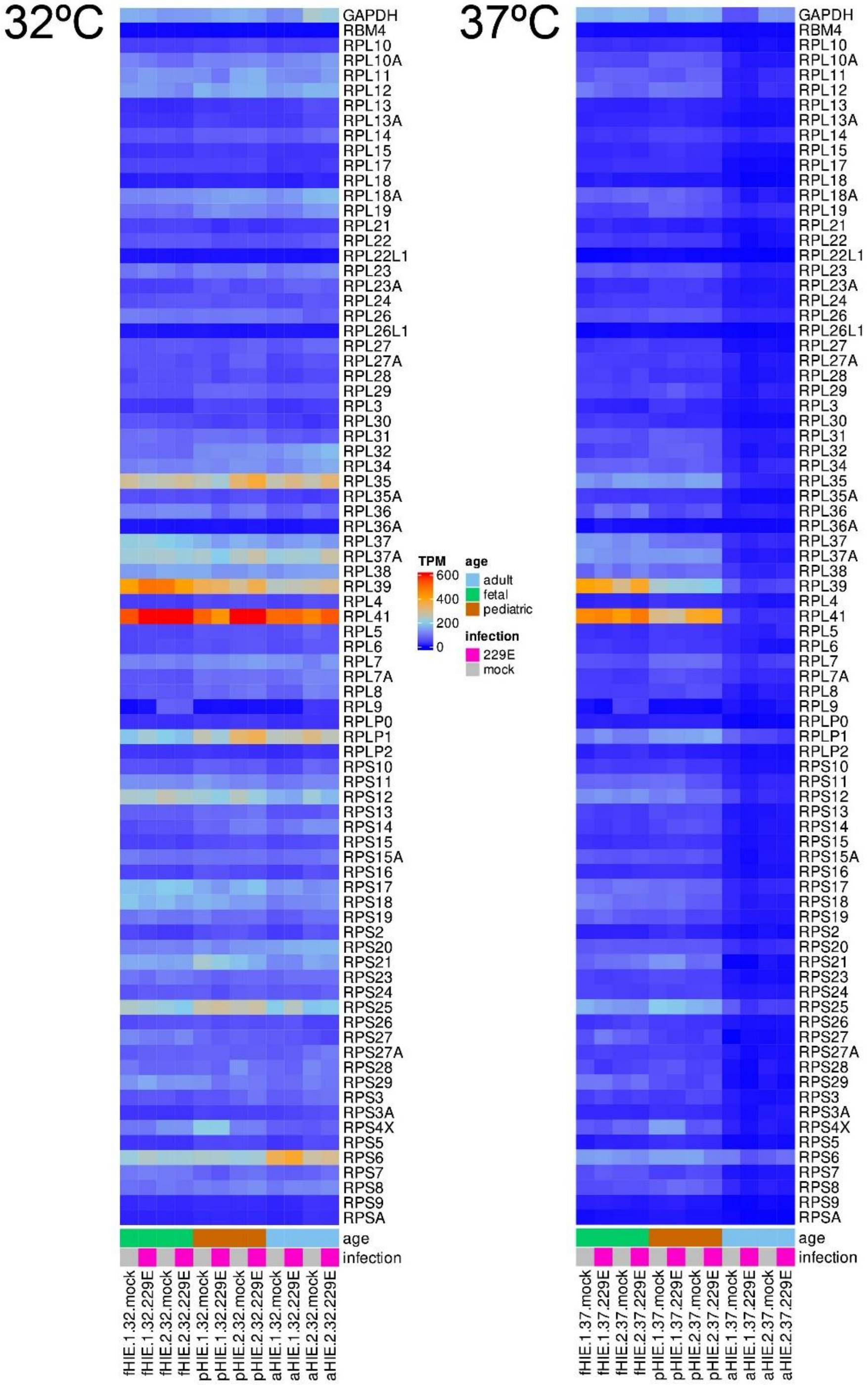

Supplementary Figure S3. Comparison of the cytoplasmic translation-associated gene expression profiles in HIEs at 32ºC and 37ºC. The heatmaps show the analysis of baseline levels of the transcripts associated with the cytoplasmic translation across two thermal conditions: 32ºC (left) and 37ºC (right). Gene expression levels are represented as transcripts per million (TPM), with red indicating high expression and blue indicating low expression. The vertical bars categorize samples by age group (adult, pediatric, and fetal) and infection status (229E-infected and mock-infected) across multiple donors. GAPDH was used as a reference gene control.

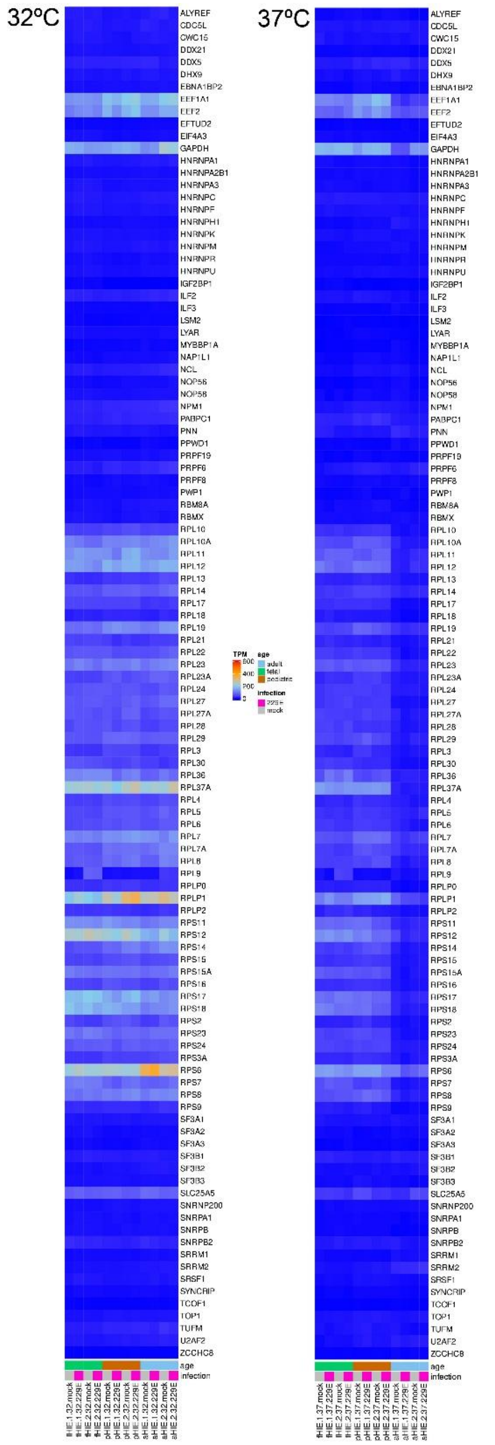

Supplementary Figure S4. Comparison of the Nop56-associated pre-rRNA complex and the spliceosome C-complex associated gene expression profiles in HIEs at 32ºC and 37ºC. The heatmaps show the analysis of baseline levels of the transcripts associated with the Nop56-associated pre-rRNA complex and the spliceosome C-complex across two thermal conditions: 32ºC (left) and 37ºC (right). Gene expression levels are represented as transcripts per million (TPM), with red indicating high expression and blue indicating low expression. The vertical bars categorize samples by age group (adult, pediatric, and fetal) and infection status (229E-infected and mock-infected) across multiple donors. GAPDH was used as a reference gene control.

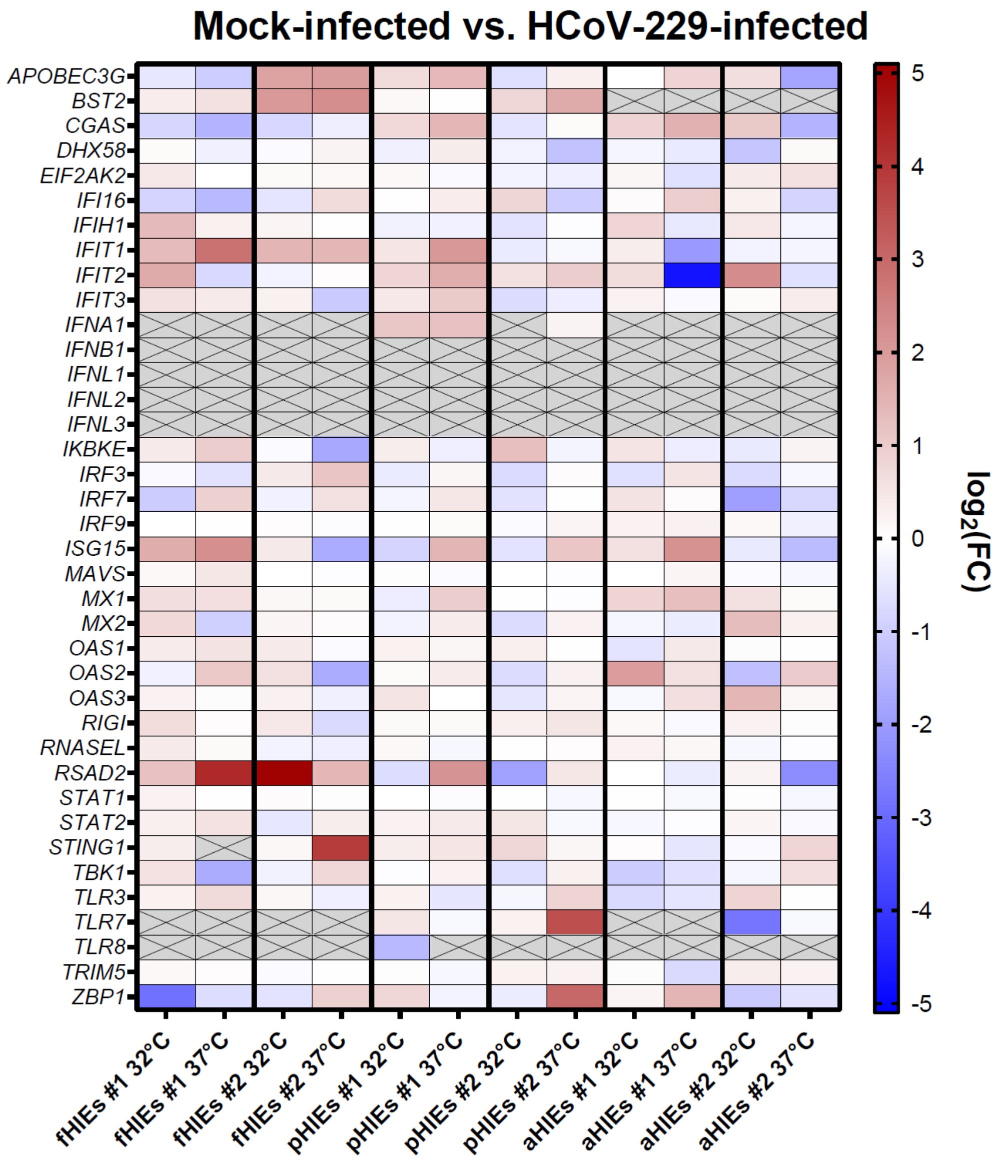

Supplementary Figure S5. Temperature-dependent modulation of innate immune gene expression in HCoV-229E-infected HIEs (mock-infected vs. virus-infected). Heatmap representation of differentially expressed genes (DEGs) associated with innate immunity and antiviral response pathways. Data are presented as log_2(FC) (Fold Change) for adult (aHIEs), pediatric (pHIEs), and fetal (fHIEs) donors (columns) across two thermal conditions: 32ºC and 37ºC. "X" indicates genes excluded from differential expression analysis due to low or zero counts, resulting in no reported value.

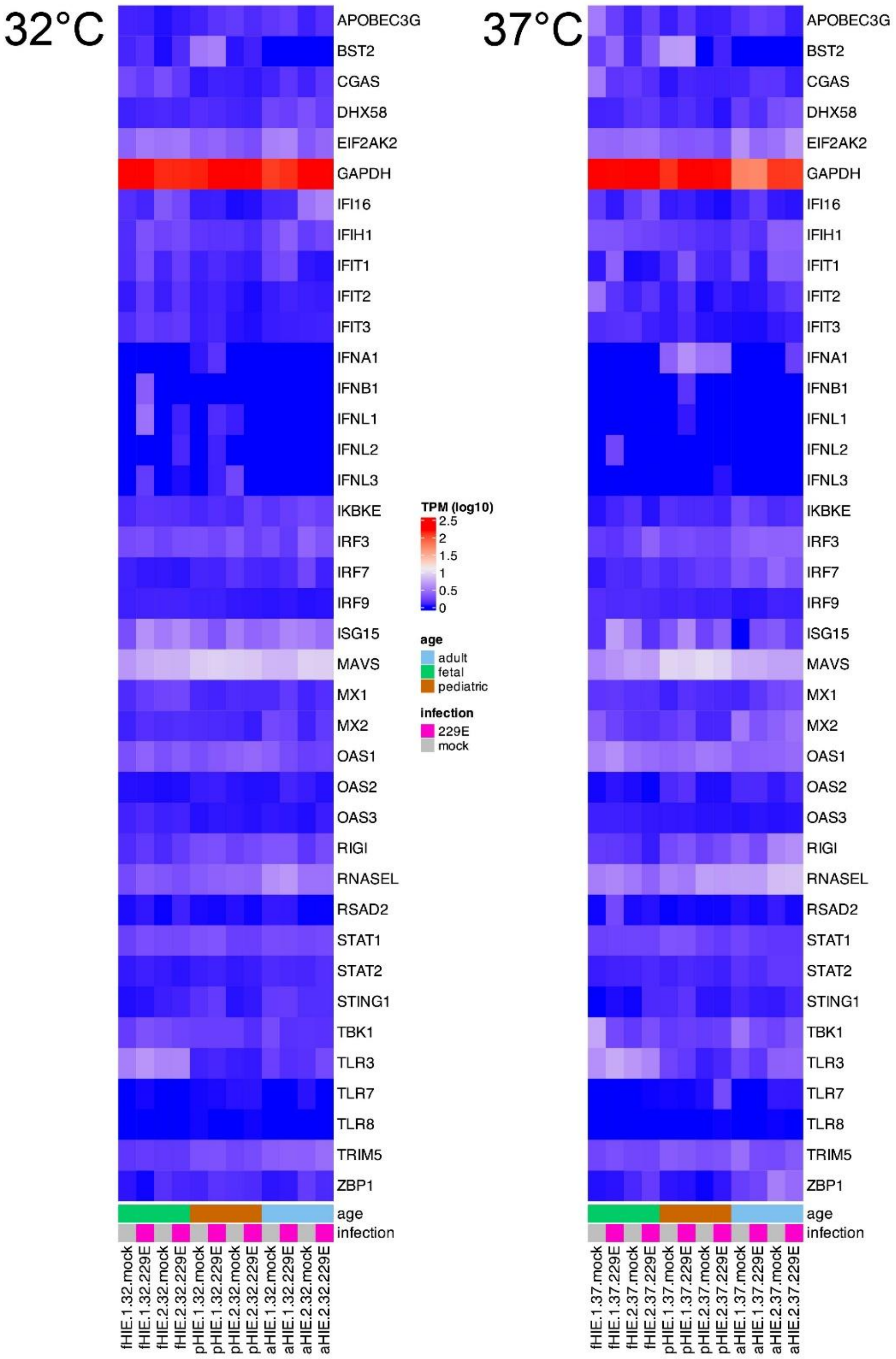

Supplementary Figure S6. **Comparison of innate immune gene expression profiles in HIEs at 32ºC and 37ºC.** The heatmaps show the analysis of baseline levels of the transcripts associated with the innate immunity and antiviral signaling across two thermal conditions: 32ºC (left) and 37ºC (right). Gene expression levels are represented as log10 transcripts per million (TPM), with red indicating high expression and blue indicating low expression. The vertical bars categorize samples by age group (adult, pediatric, and fetal) and infection status (229E-infected and mock-infected) across multiple donors. GAPDH was used as a reference gene control.

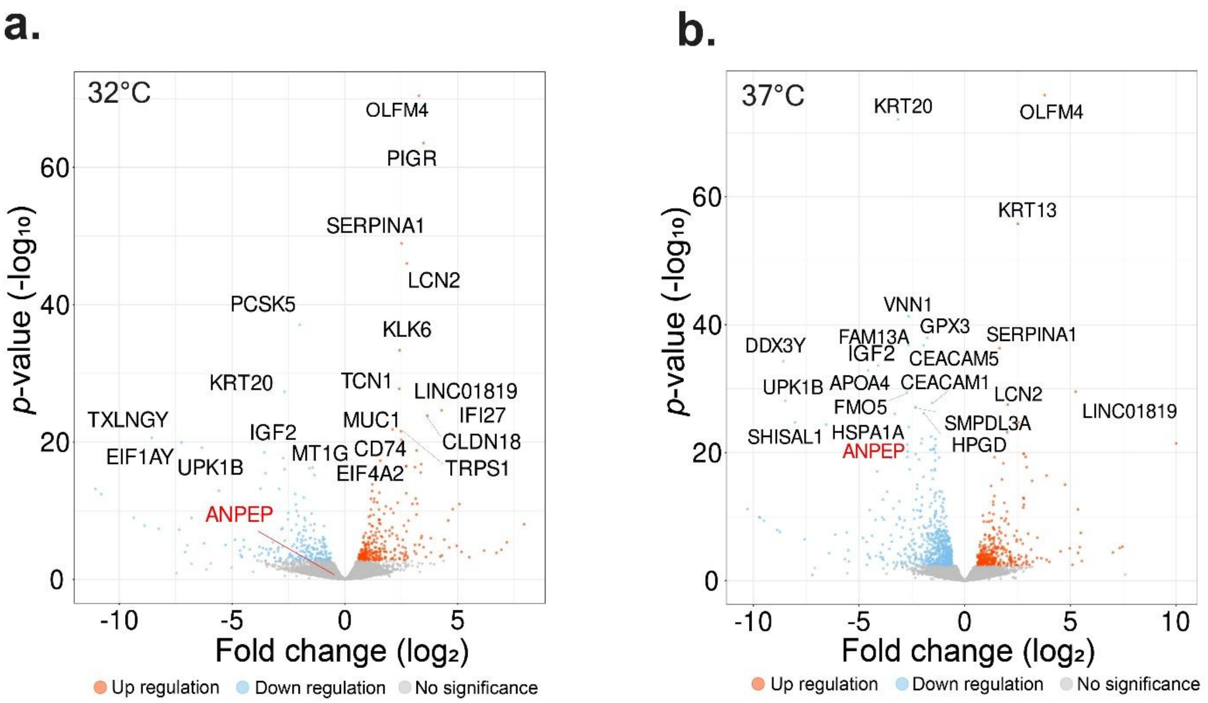

Supplementary Figure S7. Temperature-dependent transcriptomic divergence between pHIEs #1 and pHIEs #2. Volcano plots illustrate DEG profiles comparing pHIEs #1 and pHIEs #2 at (**a**) 32°C and (**b**) 37°C, based on RNA-seq data. The y-axis represents statistical significance as p-value ($-log_{10}$), while the x-axis displays the fold change (log_2), with positive values indicating higher expression in pHIEs #2 relative to pHIEs #1. Genes meeting the significance threshold of $P < 0.05$ are highlighted, and the 20 most significantly differentially expressed genes in each condition are labeled to identify the primary drivers of inter-individual variation. Additionally, the entry receptor *ANPEP* is specifically annotated to denote its expression variance between the two donors across both thermal environments.

Supplementary Table S1. List of HIEs used in this study.

Age group/Estimated gestational age	Case	Name used in this study	Source
Fetal (19 weeks)	1112	fHIEs #1	Konnikova Lab Biorepository, Yale University, United States
Fetal (23 weeks)	1136	fHIEs #2	Konnikova Lab Biorepository, Yale University, United States
Fetal (16 weeks)	210087	fHIEs #3	Amsterdam University Medical Center
Fetal (19 weeks)	210088	fHIEs #4	Amsterdam University Medical Center
Pediatric (6 years old)	T354	pHIEs #1	Department of Pediatrics, University of Cambridge, United Kingdom
Pediatric (7 years old)	T363	pHIEs #2	Department of Pediatrics, University of Cambridge, United Kingdom
Adult (51 years old)	Prep ht-104-I	aHIEs #1	Sigma-Aldrich (Product #: SCC337)
Adult (19 years old)	Prep ht-105-I	aHIEs #2	Sigma-Aldrich (Product #: SCC339)

Supplementary Table S2. List of primers and probes used in this study.

Target	Sense/Antisense/Probe	Sequence (5' → 3')	Working concentration [nM]	Reference
HCoV-229E nucleocapsid	Sense	GTTGTGGCCAATGGTGTTAAAG	600	In house
HCoV-229E nucleocapsid	Antisense	AGTGTTGCCTGACTCTTTGG	600	In house
HCoV-229E nucleocapsid	Probe	FAM-ACAATTTGCTGAGCTTGTGCCGTC-TAMRA	300	In house
HCoV-NL63 nucleocapsid	Sense	AAACCTCGTTGGAAGCGTGT	600	In house
HCoV-NL63 nucleocapsid	Antisense	CTGTGGAAAACCTTTGGCATC	600	In house
HCoV-NL63 nucleocapsid	Probe	FAM-ATGTTATTCAGTGCTTTGGTCCTCGTGAT-TAMRA	300	In house
HCoV-OC43 nucleocapsid	Sense	AGCAACCAGGCTGATGTCAATACC	600	In house
HCoV-OC43 nucleocapsid	Antisense	AGCAGACCTTCCTGAGCCTTCAAT	600	In house
HCoV-OC43 nucleocapsid	Probe	FAM-TGACATTGTCGATCGGGACCCAAGTA-TAMRA	300	In house
HCoV-HKU1 nucleocapsid	Sense	CTGGTACGATTTTGCCTCAA	600	In house
HCoV-HKU1 nucleocapsid	Antisense	ATTATTGGGTCCACGTGATTG	600	In house
HCoV-HKU1 nucleocapsid	Probe	FAM-TTGAAGGCTCAGGAAGGTCTGCTTCTAA-TAMRA	300	In house
HCoV-229E sg mRNA (25-up leader)	Sense	CTTAAGTACCTTATCTATCTACAGATAG	500	[57]
HCoV-229E sg mRNA (25900-down)	Antisense	TGACAAATCCACCCGTTTGCCCT	500	[57]
HCoV-229E sg mRNA (25820-down)	Antisense	GTCTTTCTTGTTGATGGGTACC	500	[57]
GAPDH	Sense	AGTCCTTCCACGATACCAAAG	500	In house
GAPDH	Antisense	CATGAGAAGTATGACAACAGC	500	In house

Degenerate nucleotides are as follows: Y = C or T; R = A or G; B = not A; N = any.
FAM - 6-carboxyfluorescein, TAMRA - 6-carboxy-tetramethylrhodamine

Supplementary Table S3. List of antibodies and dyes used in this study.

Antibody	Provider	Catalog number	Immunostaining dilution	Flow Cytometry dilution
Rabbit anti-Human alphacoronavirus-229E nucleocapsid	Sino Biological	40640-T62	1:400	1:1000
Mouse anti-dsRNA (clone rJ2)	Sigma-Aldrich	MABE1134	1:60	Not used
Mouse anti-villin	Santa Cruz Biotechnology	SC-58897	1:100	1:250
Mouse anti-Mucin-2	Thermo Fisher Scientific	MA5-12345	1:100	1:250
Mouse anti-Lysozyme	Thermo Fisher Scientific	MA5-28578	1:100	1:250
Mouse anti-Chromogranin A	Thermo Fisher Scientific	MA5-13096	1:100	1:250
Mouse IgG isotype control	Genetex	GTX35009	1:100	1:250
Donkey IgG anti-rabbit Alexa Fluor 488	Thermo Fisher Scientific	A-21206	1:400	1:2,000
Donkey IgG anti-mouse Alexa Fluor 594	Thermo Fisher Scientific	A-21203	1:400	1:2,000
Goat IgG anti-mouse ATTO 633	Sigma-Aldrich	78102	1:400	1:2,000
Goat IgG anti-rabbit ATTO 633	Sigma-Aldrich	41176	1:400	1:2,000
F-actin phalloidin Alexa Fluor 647	Thermo Fisher Scientific	A22287	1:400	Not used
F-actin phalloidin Alexa Fluor 546	Thermo Fisher Scientific	A22287	1:400	Not used

Legends for Datasets S1-S4

Dataset S1. RNA-seq differential expression analysis of mock-infected HIEs at 32ºC vs 37ºC.

Dataset S2. RNA-seq differential expression analysis of mock-infected HIEs vs 229E-infected HIEs.

Dataset S3. Gene Ontology (GO) enrichment analysis of genes differentially expressed between 32°C and 37°C conditions.

Dataset S4. Gene Ontology (GO) enrichment analysis of genes differentially expressed between pediatric donors.

1 Konnikova, L., Boschetti, G., Rahman, A., Mitsialis, V., Lord, J., Richmond, C., Tomov, V. T., Gordon, W., Jelinsky, S., Canavan, J., Liss, A., Wall, S., Field, M., Zhou, F., Goldsmith, J. D., Bewtra, M., Breault, D. T., Merad, M. & Snapper, S. B. High-dimensional immune phenotyping and transcriptional analyses reveal robust recovery of viable human immune and epithelial cells from frozen gastrointestinal tissue. *Mucosal Immunol* **11**, 1684-1693 (2018). https://doi.org:10.1038/s41385-018-0047-y

2 Ettayebi, K., Crawford, S. E., Murakami, K., Broughman, J. R., Karandikar, U., Tenge, V. R., Neill, F. H., Blutt, S. E., Zeng, X.-L., Qu, L., Kou, B., Opekun, A. R., Burrin, D., Graham, D. Y., Ramani, S., Atmar, R. L. & Estes, M. K. Replication of human noroviruses in stem cell–derived human enteroids. *Science* **353**, 1387-1393 (2016). https://doi.org:doi:10.1126/science.aaf5211

3 Roodsant, T., Navis, M., Aknouch, I., Renes, I. B., van Elburg, R. M., Pajkrt, D., Wolthers, K. C., Schultsz, C., van der Ark, K. C. H., Sridhar, A. & Muncan, V. A Human 2D Primary Organoid-Derived Epithelial Monolayer Model to Study Host-Pathogen Interaction in the Small Intestine. *Frontiers in Cellular and Infection Microbiology* **Volume 10 - 2020** (2020). https://doi.org:10.3389/fcimb.2020.00272

4 García-Rodríguez, I., van Eijk, H., Koen, G., Pajkrt, D., Sridhar, A. & Wolthers, K. C. Parechovirus A Infection of the Intestinal Epithelium: Differences Between Genotypes A1 and A3. *Front Cell Infect Microbiol* **11**, 740662 (2021). https://doi.org:10.3389/fcimb.2021.740662

5 Dei, A., Calitz, C., Korsten, J., Johannesson, N., Freeze, E., Eaves, A., Louis, S., Conder, R. K., Chang, W., Pajkrt, D., Wolthers, K. C., Sridhar, A. & Simmini, S. A Human Biomimetic Intestinal Mucosa Model to Study Gastrointestinal Development and Disease. *bioRxiv*, 2024.2008.2020.608742 (2024). https://doi.org:10.1101/2024.08.20.608742

6 Lie, L. K., Synowiec, A., Mazur, J., Rabalski, L. & Pyrć, K. An engineered A549 cell line expressing CD13 and TMPRSS2 is permissive to clinical isolate of human coronavirus 229E. *Virology* **588**, 109889 (2023). https://doi.org:https://doi.org/10.1016/j.virol.2023.109889

7 Schelle, B., Karl, N., Ludewig, B., Siddell, S. G. & Thiel, V. Selective replication of coronavirus genomes that express nucleocapsid protein. *J Virol* **79**, 6620-6630 (2005). https://doi.org:10.1128/jvi.79.11.6620-6630.2005

8 Pyrc, K., Jebbink, M. F., Berkhout, B. & van der Hoek, L. Genome structure and transcriptional regulation of human coronavirus NL63. *Virol J* **1**, 7 (2004). https://doi.org:10.1186/1743-422x-1-7

9 An integrated encyclopedia of DNA elements in the human genome. *Nature* **489**, 57-74 (2012). https://doi.org:10.1038/nature11247

10 Kagda, M. S., Lam, B., Litton, C., Small, C., Sloan, C. A., Spragins, E., Tanaka, F., Whaling, I., Gabdank, I., Youngworth, I., Strattan, J. S., Hilton, J., Jou, J., Au, J., Lee, J.-W., Andreeva, K., Graham, K., Lin, K., Simison, M., Jolanki, O., Sud, P., Assis, P., Adenekan, P., Miyasato, S., Zhong, W., Luo, Y., Myers, Z., Cherry, J. M. & Hitz, B. C. Data navigation on the ENCODE portal. *Nature Communications* **16**, 9592 (2025). https://doi.org:10.1038/s41467-025-64343-9

11 Bray, N. L., Pimentel, H., Melsted, P. & Pachter, L. Near-optimal probabilistic RNA-seq quantification. *Nature Biotechnology* **34**, 525-527 (2016). https://doi.org:10.1038/nbt.3519

12 Soneson, C., Love, M. & Robinson, M. Differential analyses for RNA-seq: transcript-level estimates improve gene-level inferences [version 2; peer review: 2 approved]. *F1000Research* **4** (2016). https://doi.org:10.12688/f1000research.7563.2

13 Love, M. I., Huber, W. & Anders, S. Moderated estimation of fold change and dispersion for RNA-seq data with DESeq2. *Genome Biology* **15**, 550 (2014). https://doi.org:10.1186/s13059-014-0550-8

14 Anders, S. & Huber, W. Differential expression analysis for sequence count data. *Genome Biology* **11**, R106 (2010). https://doi.org:10.1186/gb-2010-11-10-r106
15 Yu, G., Wang, L. G., Han, Y. & He, Q. Y. clusterProfiler: an R package for comparing biological themes among gene clusters. *Omics* **16**, 284-287 (2012). https://doi.org:10.1089/omi.2011.0118
16 Wu, T., Hu, E., Xu, S., Chen, M., Guo, P., Dai, Z., Feng, T., Zhou, L., Tang, W., Zhan, L., Fu, X., Liu, S., Bo, X. & Yu, G. clusterProfiler 4.0: A universal enrichment tool for interpreting omics data. *The Innovation* **2**, 100141 (2021). https://doi.org:https://doi.org/10.1016/j.xinn.2021.100141
17 Chen, H.-C. The Dynamic Linkage between Provirus Integration Sites and the Host Functional Genome Property Alongside HIV-1 Infections Associated with Antiretroviral Therapy. *Vaccines* **11**, 402 (2023).
18 Schindelin, J., Arganda-Carreras, I., Frise, E., Kaynig, V., Longair, M., Pietzsch, T., Preibisch, S., Rueden, C., Saalfeld, S., Schmid, B., Tinevez, J. Y., White, D. J., Hartenstein, V., Eliceiri, K., Tomancak, P. & Cardona, A. Fiji: an open-source platform for biological-image analysis. *Nat Methods* **9**, 676-682 (2012). https://doi.org:10.1038/nmeth.2019
